# Rationale, Design, and the Baseline Characteristics of the RHDGen (The Genetics of Rheumatic Heart Disease) Network Study†

**DOI:** 10.1161/CIRCGEN.121.003641

**Published:** 2022-12-22

**Authors:** Tafadzwa Machipisa, Chishala Chishala, Gasnat Shaboodien, Liesl J. Zühlke, Babu Muhamed, Shahiemah Pandie, Jantina de Vries, Nakita Laing, Alexia Joachim, Rezeen Daniels, Mpiko Ntsekhe, Christopher T. Hugo-Hamman, Bernard Gitura, Stephen Ogendo, Peter Lwabi, Emmy Okello, Albertino Damasceno, Celia Novela, Ana O. Mocumbi, Geoffrey Madeira, John Musuku, Agnes Mtaja, Ahmed ElSayed, Huda H.M. Alhassan, Fidelia Bode-Thomas, Christopher Yilgwan, Ganiyu Amusa, Esin Nkereuwem, Nicola Mulder, Raj Ramesar, Maia Lesosky, Heather J. Cordell, Michael Chong, Bernard Keavney, Guillaume Paré, Mark E. Engel

**Affiliations:** Department of Medicine, University of Cape Town and Groote Schuur Hospital, Cape Town, South Africa (T.M., C.C., G.S., L.J.Z., B.M., S.P.; J.d.V., N.L., A.J., R.D., M.N., M.E.E.).; Department of Medicine, Cape Heart Institute, University of Cape Town, Cape Town, South Africa (T.M., G.S., L.J.Z., B.M., M.E.E.).; Population Health Research Institute, Hamilton, ON, Canada (T.M., B.M., M.C., G.P.).; Thrombosis and Atherosclerosis Research Institute, David Braley Cardiac, Vascular and Stroke Research Institute, Hamilton, ON, Canada (T.M., B.M., M.C., G.P.).; Department of Pathology and Molecular Medicine, Michael G. DeGroote School of Medicine, Hamilton, ON, Canada (T.M., B.M., M.C., G.P.).; Division of Cardiology, University of KwaZulu-Natal, Msunduzi, KwaZulu-Natal (C.C.).; Division of Pediatric Cardiology, Department of Pediatrics and Child Health, Red Cross War Memorial Children’s Hospital, Cape Town, South Africa (L.J.Z.).; South African Medical Research Council, Extramural Research and Internal Portfolio, Cape Town, South Africa (L.J.Z.).; Rheumatic Heart Disease Clinic, Windhoek Central Hospital, Ministry of Health and Social Services, Windhoek, Republic of Namibia (C.T.H.-H.).; Cardiology Department of Medicine, Kenyatta National Hospital, University of Nairobi, Nairobi, Kenya (B.G.).; Uganda Heart Inst, Departments of Adult and Pediatric Cardiology, Kampala, Uganda (S.O.).; School of Medicine, Maseno Univ, Kenya (P.L., E.O.).; Faculty of Medicine, Eduardo Mondlane Univ/Nucleo de Investigaçao, Departamento de Medicina, Hospital Central de Maputo, Maputo, Mozambique (A.D., C.N.).; Instituto Nacional de Saúde Ministério da Saúde, Mozambique (A.O.M.).; WHO Mozambique, Maputo, Mozambique (G.M.).; University Teaching Hospital, Children’s Hospital, University of Zambia, Lusaka, Zambia (J.M., A.M.).; Department of Cardiothoracic Surgery, Alshaab Teaching Hospital, Alazhari Health Research Centre, Alzaiem Alazhari University, Khartoum, Sudan (A.E., H.H.M.A.).; Deptartments of Pediatrics and Medicine, Jos University Teaching Hospital and University of Jos, Jos, Plateau State, Nigeria (F.B.-T., C.Y., G.A., E.N.).; Computational Biology Division, Department of Integrative Biomedical Sciences, Institute of Infectious Disease and Molecular Medicine, Faculty of Health Sciences (N.M.), University of Cape Town, Cape Town, South Africa.; Department of Pathology (R.R.), University of Cape Town, Cape Town, South Africa.; Division of Epidemiology and Biostatistics, School of Public Health and Family Medicine (M.L.), University of Cape Town, Cape Town, South Africa.; Population Health Sciences Institute, Faculty of Medical Sciences, Newcastle University, International Centre for Life, Newcastle upon Tyne, UK (H.J.C.).; Division of Cardiovascular Sciences, School of Medical Sciences, Faculty of Biology, Medicine and Health, The University of Manchester, UK (B.K.).; Manchester University NHS Foundation Trust, Manchester Academic Health Science Centre, UK (B.K.).; Department of Clinical Epidemiology and Biostatistics, McMaster University, Hamilton, ON, Canada (G.P.).

**Keywords:** cardiovascular diseases, genetics, infections, rheumatic heart disease

## Abstract

The genetics of rheumatic heart disease (RHDGen) Network was developed to assist the discovery and validation of genetic variations and biomarkers of risk for rheumatic heart disease (RHD) in continental Africans, as a part of the global fight to control and eradicate rheumatic fever/RHD. Thus, we describe the rationale and design of the RHDGen study, comprising participants from 8 African countries.

**Methods:**

RHDGen screened potential participants using echocardiography, thereafter enrolling RHD cases and ethnically-matched controls for whom case characteristics were documented. Biological samples were collected for conducting genetic analyses, including a discovery case-control genome-wide association study (GWAS) and a replication trio family study. Additional biological samples were also collected, and processed, for the measurement of biomarker analytes and the biomarker analyses are underway.

**Results:**

Participants were enrolled into RHDGen between December 2012 and March 2018. For GWAS, 2548 RHD cases and 2261 controls (3301 women [69%]; mean age [SD], 37 [16.3] years) were available. RHD cases were predominantly Black (66%), Admixed (24%), and other ethnicities (10%). Among RHD cases, 34% were asymptomatic, 26% had prior valve surgery, and 23% had atrial fibrillation. The trio family replication arm included 116 RHD trio probands and 232 parents.

**Conclusions:**

RHDGen presents a rare opportunity to identify relevant patterns of genetic factors and biomarkers in Africans that may be associated with differential RHD risk. Furthermore, the RHDGen Network provides a platform for further work on fully elucidating the causes and mechanisms associated with RHD susceptibility and development.

Rheumatic heart disease (RHD) is a preventable sequela of rheumatic fever, characterized by permanent heart valve damage.^1^ RHD is the leading indication for cardiac surgery in the young (adolescents and young adults) in Sub-Saharan Africa, which carries a quarter of the global disease burden.^1–3^ Worldwide, RHD affects ≈40.5 million individuals, claiming up to 340 000 lives annually,^4^ the majority of whom live in low- and middle-income countries. The recognition of RHD as a common cause of heart failure, infective endocarditis, stroke, and maternal and perinatal mortality over the past 2 decades led to the reinstatement of RHD as a major public health priority.^3^ In 2018, the World Health Assembly passed a resolution on RHD mandating a coordinated global response.^1,5,6^

RHD’s persistence as a major public health issue in low- and middle-income countries is attributed to associated risk factors such as the lack of effective RHD prevention, control and elimination initiatives. Additionally, known risk factors for rheumatic fever (RHD’s prequel’s; eg, failure to drastically change socioeconomic factors [affecting those living in poverty and overcrowded dwellings], limited use/availability of antibiotics [especially, intramuscular Penicillin G Benzathine], and increased genetic susceptibility^7^) may also play a role. A recent systematic review indicated that rheumatic fever was heritable, reporting high odds of rheumatic fever among monozygotic twins with an estimated heritability of 60%.^8^ Furthermore, 60% of rheumatic fever patients develop RHD, and despite there being a proven association between group A *Streptococcus* infection and RHD, the triggered autoimmune process in RHD can occur autonomously after removing the stimulus. This suggests that after initiation of the autoimmune response via molecular mimicry, host factors (most likely genetic) play an important role in disease progression in susceptible individuals.^9–11^ Thus, using genetic studies, which could identify people at high risk, may help develop effective RHD prevention/control measures such as targeted approaches for RHD screening and treatments.^8,12^ Utilizing a hypothesis-free approach, such as a genome-wide association study (GWAS), may highlight key genetic risk factors associated with disease susceptibility.^13^

Here, we report on the rationale and design, and present baseline characteristics, of the Rheumatic Heart Disease Genetics (RHDGen) study with the primary objective to identify genetic variants affecting susceptibility and resistance to RHD in Africans. The secondary objective is to convey the polygenic nature of RHD susceptibility, its heritability in the study population, and to replicate any prior or novel GWAS findings. While seeking to identify genetic risk factors associated with RHD, and elucidate the pathogenesis of RHD susceptibility in Africans, a biorepository was formed, which will also serve as a resource for further studies, including biomarker analyses.

## Methods

**Figure 1.**
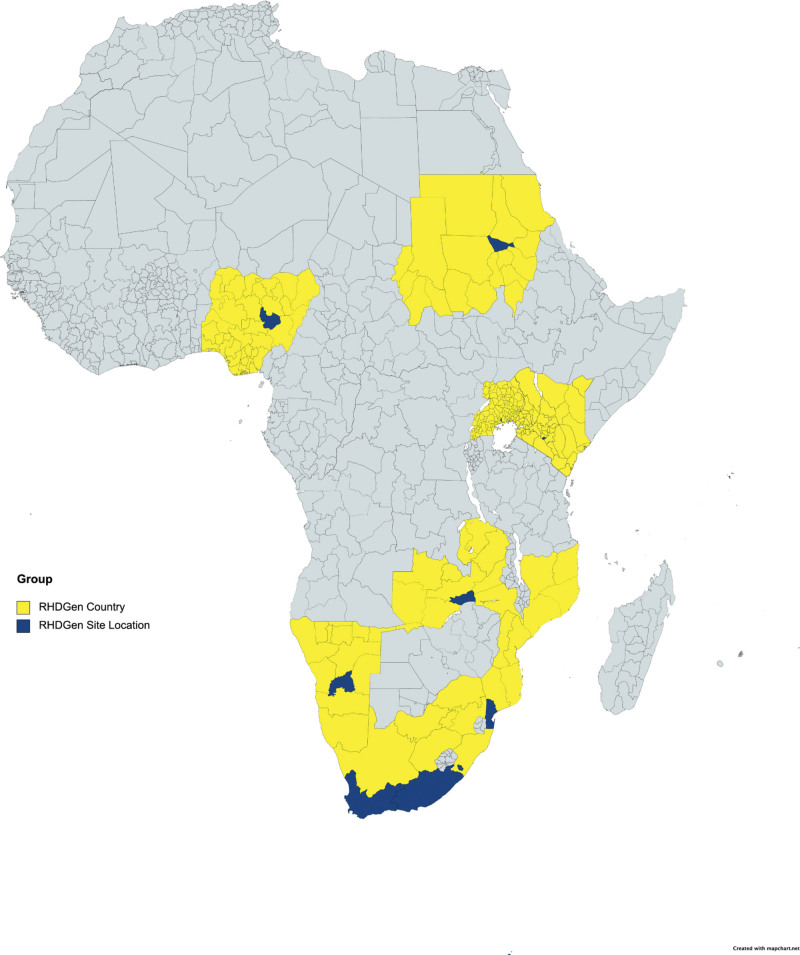
Location of the Rheumatic Heart Disease Genetics (RHDGen) recruitment site countries (yellow) and regional sites (dark blue).

The development of the RHDGen Network (participating countries and sites: Figure 1 and Tables 1 and 2) and its related substudies was approved by appropriate institutional review committees, and all subjects provided written informed consent. Full details of data and methods used in this study are presented in the Supplemental Material and Methods. As per the American Heart Association’s TOP (Transparency and Openness Promotion) Guidelines, we declare that upon reasonable request data will be made available. Due to the sensitive nature of the data collected for this study, reasonable requests to access the dataset from qualified researchers trained in human subject confidentiality protocols may be sent to mark.engel@uct.ac.za and cc to taffymach@yahoo.com. The authors declare that all other supporting data are available within the article and the Supplemental Material.

**Table 1.**
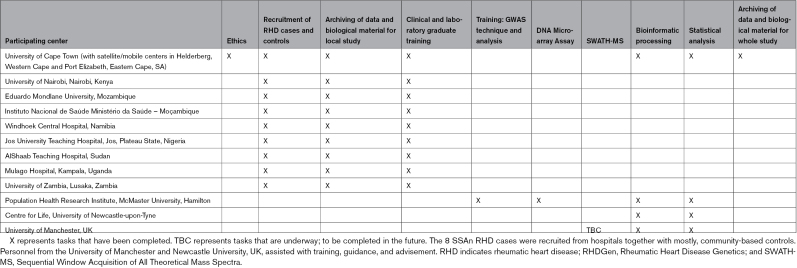
Participating Centers for RHDGen and Scheduled Activities

**Table 2.**
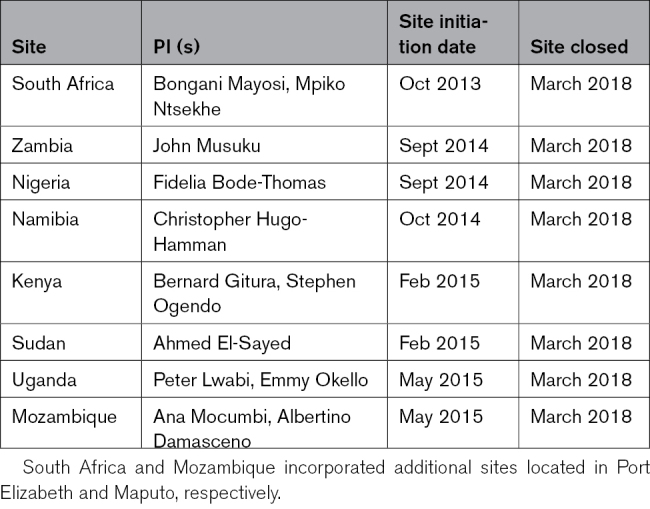
RHDGen Countries, their Principal Investigator (PI), and Site Timelines

## Results

### Study Timeline and Baseline Clinical Characteristics

**Figure 2.**
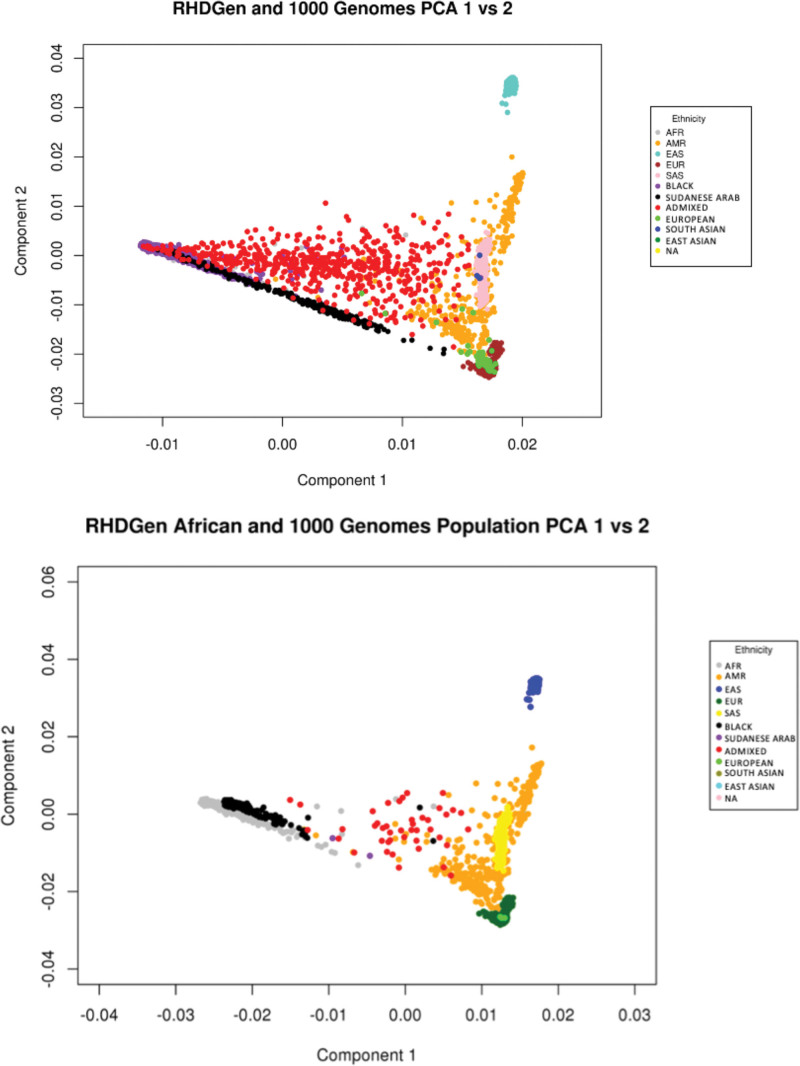
**An example of the principal component analysis (PCA) from GCTA for the participants of the RHDGen GWAS and the trio family study.** *NB*: 1000G reference populations were from AFR (Black and African American), AMR (Ad Mixed American), EAS (East Asian), EUR (European) and SAS (South Asian) ancestries. From the 8 Sub-Saharan African countries in RHDGen, all populations self-reported as citizens/permanent residents of an African country. Hence, the “BLACK” group represents Black Africans, “ADMIXED” represents the “South African Coloureds” (SAC; ie, the same as the “Admixed Africans” description), “SUDANESE ARAB” are those who reside in Sudan and self-report being of Arab descent/ethnicity, “EUROPEAN” are those of European ancestry, “SOUTH ASIAN” are those of South Asian ancestry, “EAST ASIAN” are those of East Asian ancestry, and “NA” are those who did not self-report as identifying with any ethnicity/known ancestry.

From December 31, 2012 to March 31, 2018, all GWAS participants and trio probands were screened by echocardiography. Preliminary data cleaning and analyses commenced May 15, 2017, until June 30, 2020. The GWAS arm included 2548 RHD cases and 2261 controls (3301 women [69%]; mean [SD] age, 37 [16.3] years), as per Table 3. RHD cases recruited were predominantly Black (66%), Admixed (23%), and other ethnicities classified as continental African citizens (11%); principal component analysis presented in Figure 2A. Baseline characteristics which included 34% of cases were asymptomatic, 26% of cases had valve surgery, and 23% of cases had atrial fibrillation. Most cases (41%) had a slight limitation in physical activity categorized by the New York Heart Association functional classification (Class II); presenting with mild symptoms (mild shortness of breath and/or angina) and slight limitation during ordinary activity.^14^ The trio family-based arm included 116 RHD trio probands and 232 matching parents; from the different ancestries across the 8 Sub-Saharan Africa countries in RHDGen (Figure 2B).

**Table 3.**
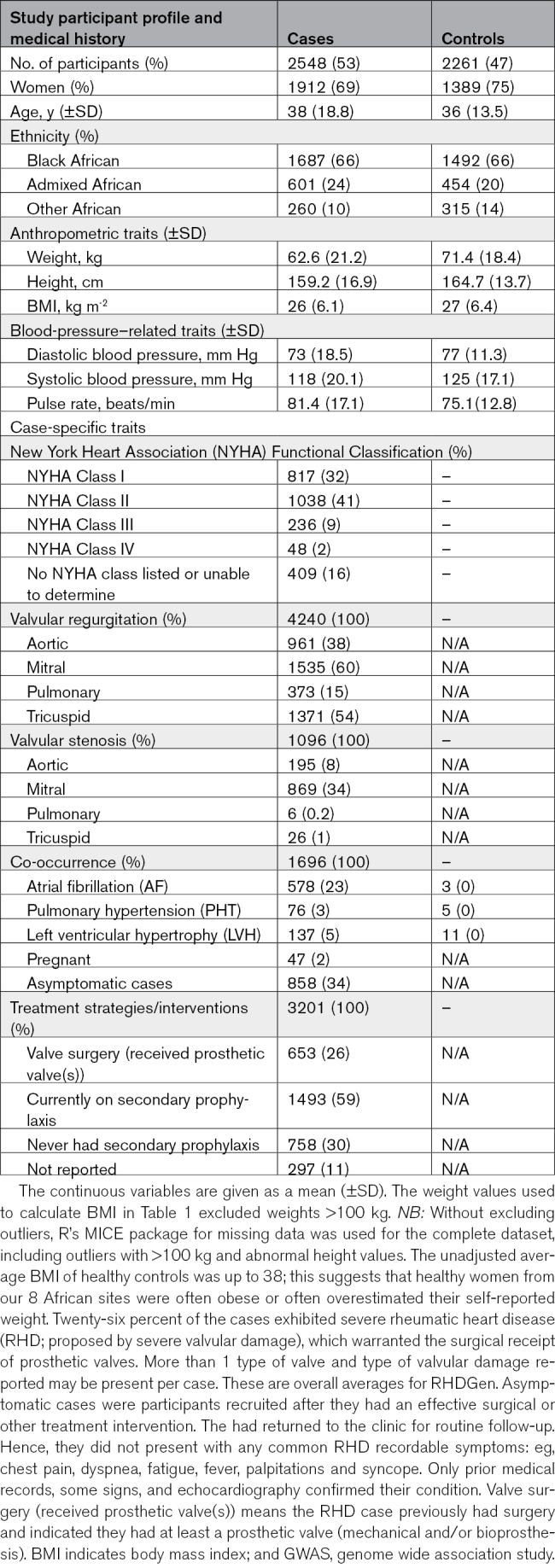
Clinical Characteristics of GWAS Participants

## Incidental Outcomes and Benefits

The RHDGen Network was part of the ASAP programme for ARF/RHD in Africa, which incorporates awareness-raising as 1 of 4 pillars to succesful eradication of RHD from Africa.^15^ Thus, fundamental to the RHDGen project is an overarching commitment to addressing the needs of this patient community in the different countries in which the research took place. Thus, a crucial component of our work is research aimed at making an impact as driven by the African Union communique.^16^

Thus far, the RHDGen Network has also developed additional indirect patient and community benefits, including increased access to RHD screening and patient care, developed and participated in patient/engagement events (eg, Listen to your heart).^17^ Hosted disease/genetics educational awareness activities, trained several experts in the field, as well as, advocated globally to change RHD from a neglected tropical disease status to a global health priority. In particular, our work on this and other RHD-related research projects has allowed us to work with WHO Africa to revise treatment guidelines for RHD in Africa. Furthermore, this research has allowed our research teams to engage with the World Heart Federation to lobby for greater focus on RHD and other heart conditions prevalent in the Global South.

## Discussion

RHDGen is one of the largest case-control genetic association studies for RHD to date. It is also one of the first studies to evaluate the genetic susceptibility of RHD in continental Africans. While other genetic association studies have examined RHD in 4 other populations (Aboriginal Australians, Asians, Europeans, and Oceanians^18–20^), RHDGen is the first to do so in multiple African countries at a large scale.^21^

RHDGen had several unique and versatile features in its rationale, design, and execution. First, a resourceful aspect of this study was that the case report form was developed to include the majority of fields from other large RHD studies, like REMEDY (Global Rheumatic Heart Disease Registry). This allowed both current and future clinical/epidemiological and genetic questions to be addressed in the current study, as well as further research, such as meta-analyses with other epidemiological studies. Second, the presence of REMEDY IDs and participants may allow future prospective case analyses, as both participants are screened during REMEDY and RHDGen, as well as retrospective case-control analyses can be performed and genetic factors explored, too. Third, the transfer of the paper-based case report form to the electronic case report form in OpenClinica helped with real-time access to data and actionable visualizations, which made the dataset cleaner and improved workflows. Subsequently, another resourceful aspect of this study was the development of the RHD biobank, as it is a useful resource for future RHD work in Africans. Finally, another innovative aspect of this study is to use family-based genetic data for replication to minimize false positive rates.

RHDGen had several context-specific challenges due to using a family-based design for replication. Although family studies were previously the gold standard for genetic research due to their robustness, in practice they are now rarely used. This is due to the various challenges associated with recruiting complete families, often leading to smaller sample sizes than targeted.^22^ Similarly, RHDGen’s trio family recruitment numbers were significantly lower than anticipated; with only 440 families before QC (including parent–child duo families) versus the anticipated 2000. In our study across 8 different African countries, most issues arose from missing parental information.

Key factors that made complete trio recruitment difficult in RHDGen were: First, the African country with the largest GWAS recruitment numbers (ie, South Africa), had mostly female-headed households (57%) and elevated father absenteeism (≈14%) from a variety of reasons including possibly the high adult deaths from HIV/AIDs, since the 1980s. Second, trio probands were estranged from parents who worked as migrant workers, due to the impacts of the migrant labor system, for example, in South Africa under apartheid (1994), which prohibited male pass-controlled laborers from cohabiting with their spouse/child in work-related housing. This has been partially maintained in low income occupations, promoting offspring and working parent(s) estrangement/absenteeism.^23^ Third, distances between adult trio probands and their parents’ homes were far or inaccessible, significantly reducing dual parental recruitment. For instance, public health hospital systems and structures (eg, cardiac clinics) are often centralized and near richer neighborhoods (including even more private cardiac facilities), as CVD used to be thought to commonly affect only the rich. However, nowadays CVDs like RHD often occur in the children of the poor, who often live further away in impoverished, overcrowded townships/farm areas.

Eventually, toward the end of the study, a few community outreaches were attempted to increase trio family member recruitment. For example, Cape Town developed mobile cardiac clinic trips at Helderberg to increase trio recruitment. This outreach was a weekend mobile clinic site and a community-based outreach, enabling complete trio families to provide consent, be screened, provide blood, and be enrolled/included, all at once. Ultimately, the parents were mostly recruited in the countryside (ie, rural or farming areas); hence, these are poorly-resourced areas where improvization was needed and limited data were available. Hence, a polygenic transmission disequilibrium testing method was used for replication, to resolve the small sample size and limited parental information available.^21,24^ Thus, future family studies in Africa are recommended to include relevant contextual/tailored financial, transportation, and logistics considerations flexible enough to maximize the desired enrollment of the study participants.

## Future Directions

We plan to carry out genetic and nongenetic follow-ups for further validation and replication with RHDGen; for instance, attempting collaborative meta-analyses, and mendelian randomization. Future research initiatives in RHD research are recommended to include functional validation through gene disruption studies, fine-mapping, and additional genotyping. Furthermore, a subset of RHDGen serum samples are currently undergoing SWATH-MS evaluation, which will represent the first RHD proteomic profiles in continental Africans. Thus, we invite investigators and funders with shared interests and resources to join and collectively enhance research efforts in RHDGen.

## Conclusions

In summary, the RHDGen Network is a global collaboration among investigators who have recruited patients with RHD across Africa, seeking to gain a better understanding of RHD susceptibility, pathogenesis, and disease development. The RHDGen Network developed a unique network and biorepository to investigate RHD genetics and biomarkers in continental Africans. Ultimately, we hope that our work and collaborations can guide future RHD diagnostic, prevention, management and treatment options.

This research was funded in whole, or in part, by the Wellcome Trust [099313/B/12/A]. For the purpose of open access, the author has applied a CC BY public copyright license to any Author Accepted Article version arising from this submission.

## Article Information

### Acknowledgments

In memory of deceased co-authors Bongani M Mayosi MD DPhil, Lungile Pepeta, MD and Veronica Francis, RN.

We would like to thank participants for being part of our study and the study planning, training, data entry, and cleaning staff of the Mayosi Research Group (MRG) Coordinating Office team, Department of Medicine, UCT, and collaborating sites. We acknowledge the UCT HICRA/CHI and its cardiovascular genetics (CVG) laboratory for assisting with all the preliminary wet laboratory work. We would also like to acknowledge PHRI and its CRLB-GMEL lab for assisting with the wet and dry laboratory work. Finally, we would also like to commend the Keavney Lab, Manchester, UK for their work on the proteomics; supported by the British Heart Foundation (BHF).

BMM (before his passing), BK, GP, HJC, RR, J.D-V, ML, TM, and MEE made substantial contributions to the conception and design of the RHDGen study. BMM, CC, MEE, SP C.H-H., SO, CM, JM, A.M., A.E-S., F.B-T., NL, MN, and LZ contributed to the acquisition of study participants and samples, as well as the development, management and completion of the RHDGen participant database. TM, BM, SP, MEE, and BMM reconciled the trios. BMM, GS, BM, and TM contributed to the laboratory studies by handling, acquiring, and processing RHDGen’s biological samples and developing, managing, and completing the RHDGen biological study samples database and the RHDGen biobank at HICRA & CHI, CVG laboratory, UCT. TM, MC, MEE, and GP performed and reviewed the statistics and bioinformatics analyses. TM, GP, MC, BK, HJC, MEE, and BMM managed the study results’ interpretation. TM wrote the first draft of the article under the supervision of MEE and GP. All authors contributed to revisions and approved the final version for publication, as per the international committee of medical journal editors (ICMJE) criteria.

### Sources of Funding

*RHDGen:* The RHDGen Network was founded by funding awarded to Bongani Mayosi by the Wellcome Trust; described here: https://h3africa.org/index.php/consortium/the-rhdgen-network-genetics-of-rheumatic-heart-disease-and-molecular-epidemiology-of-streptococcus-pyogenes-pharyngitis/, https://app.dimensions.ai/details/grant/grant.3640606 and https://europepmc.org/grantfinder/grantdetails?query=pi%3A%22Mayosi%2BBM%22%2Bgid%3A%22099313%22%2Bga%3A%22Wellcome%20Trust%22. This research was funded in whole, or in part, by the Wellcome Trust [Grant number: 099313/B/12/A].

TM was supported by scholarships from the UCT (Mayosi Research Group Fellowships, the Crasnow Travel Fellowship, the Departmental Research Committee (DRC) of Medicine and the Pan African Society of Cardiology Award), Wellcome Trust, and Population Health Research Institute (PHRI) and McMaster University. LJZ Zühlke was supported by the South African Medical Research Council (SAMRC) through its Division of Research Capacity Development under the Mid-Career Scientist Programme. LJZ also received funding received from the National Research Foundation of South Africa (NRFSA), as well as the UK Medical Research Council (MRC) and the UK Department for International Development (DFID) under the MRC/DFID Concordat agreement, via the African Research Leader Award (MR/S005242/1). MC is supported by a Canadian Institute of Health Research doctoral award (# 412329) and has received Bayer’s consulting fees. GP has received consulting fees from Sanofi, Bristol-Myers Squibb, Lexicomp, Amgen, and Bayer; has received support for research through his institution from Sanofi; and has received support from the Canada Research Chair in Genetic and Molecular Epidemiology, and CISCO Professorship in Integrated Health Systems. ME received support from the RHDGen Wellcome Trust grant and the South African National Research Foundation (NRF) # 116287, the American Heart Association, United States (Grant number: NW17SFRN33630027) and UCT. BK is supported by the BHF and is a BHF Professor of Cardiovascular Medicine and the Director of the Manchester BHF Accelerator, University of Manchester.

### Disclosures

None.

### Supplemental Material

Supplemental Methods

Supplemental Outcomes

Figures S1–S6

References^25–49^

## Supplementary Material


