# Rationale, Design, and the Baseline Characteristics of the RHDGen (The Genetics of Rheumatic Heart Disease) Network Study

**DOI:** 10.1161/CIRCGEN.121.003641

**Published:** 2022-12-22

**Authors:** Tafadzwa Machipisa, Chishala Chishala, Gasnat Shaboodien, Liesl J. Zühlke, Babu Muhamed, Shahiemah Pandie, Jantina de Vries, Nakita Laing, Alexia Joachim, Rezeen Daniels, Mpiko Ntsekhe, Christopher T. Hugo-Hamman, Bernard Gitura, Stephen Ogendo, Peter Lwabi, Emmy Okello, Albertino Damasceno, Celia Novela, Ana O. Mocumbi, Geoffrey Madeira, John Musuku, Agnes Mtaja, Ahmed ElSayed, Huda H.M. Alhassan, Fidelia Bode-Thomas, Christopher Yilgwan, Ganiyu Amusa, Esin Nkereuwem, Nicola Mulder, Raj Ramesar, Maia Lesosky, Heather J. Cordell, Michael Chong, Bernard Keavney, Guillaume Paré, Mark E. Engel, for the RHDGen Network Consortium

**Affiliations:** 1 Dept of Medicine, Univ of Cape Town & Groote Schuur Hospital; School of Public Health & Family Medicine, Univ of Cape Town, Cape Town, South Africa; 2 Cape Heart Inst (CHI), Dept of Medicine, School of Public Health & Family Medicine, Univ of Cape Town, Cape Town, South Africa; 3 Population Health Rsrch Inst (PHRI), Extramural Rsrch & Internal Portfolio (VP-ERIP), Cape Town, South Africa; 4 Thrombosis & Atherosclerosis Rsrch Inst, David Braley Cardiac, Vascular & Stroke Rsrch Inst; Extramural Rsrch & Internal Portfolio (VP-ERIP), Cape Town, South Africa; 5 Dept of Pathology & Molecular Medicine, Michael G. DeGroote School of Medicine, Extramural Rsrch & Internal Portfolio (VP-ERIP), Cape Town, South Africa; 6 Division of Cardiology, Univ of KwaZulu-Natal, Msunduzi, KwaZulu-Natal; Extramural Rsrch & Internal Portfolio (VP-ERIP), Cape Town, South Africa; 7 Division of Pediatric Cardiology, Dept of Pediatrics & Child Health, Red Cross War Memorial Children’s Hospital, School of Public Health & Family Medicine, Univ of Cape Town, Cape Town, South Africa; 8 South African Medical Rsrch Council (SAMRC), Extramural Rsrch & Internal Portfolio (VP-ERIP), Cape Town, South Africa; 9 Rheumatic Heart Disease Clinic, Windhoek Central Hospital, Ministry of Health & Social Services, Windhoek, Republic of Namibia; 10 Cardiology Dept of Medicine, Kenyatta National Hospital, Univ of Nairobi, Nairobi, Kenya; 11 Uganda Heart Inst, Depts of Adult & Pediatric Cardiology, Kampala, Uganda; 12 School of Medicine, Maseno Univ, Kenya; 13 Faculty of Medicine, Eduardo Mondlane Univ / Nucleo de Investigaçao, Departamento de Medicina, Hospital Central de Maputo, Maputo, Mozambique; 14 Instituto Nacional de Saúde Ministério da Saúde – Moçambique; 15 WHO Mozambique, Maputo Mozambique; 16 Univ Teaching Hospital, Children’s Hospital, Univ of Zambia, Lusaka, Zambia; 17 Dept of Cardiothoracic Surgery, Alshaab Teaching Hospital, Alazhari Health Rsrch Ctr, Alzaiem Alazhari Univ, Khartoum, Sudan; 18 Depts of Pediatrics & Medicine, Jos Univ Teaching Hospital & Univ of Jos, Jos, Plateau State Nigeria; 19 Computational Biology Division, Dept of Integrative Biomedical Sciences, Inst of Infectious Disease & Molecular Medicine, Faculty of Health Sciences, School of Public Health & Family Medicine, Univ of Cape Town, Cape Town, South Africa; 20 Dept of Pathology, School of Public Health & Family Medicine, Univ of Cape Town, Cape Town, South Africa; 21 Division of Epidemiology & Biostatistics, School of Public Health & Family Medicine, Univ of Cape Town, Cape Town, South Africa; 22 Population Health Sciences Inst, Faculty of Medical Sciences, Newcastle Univ, International Ctr for Life, Newcastle upon Tyne Manchester Academic Health Science Ctr, UK; 23 Division of Cardiovascular Sciences, School of Medical Sciences, Faculty of Biology, Medicine & Health, The Univ of Manchester Manchester Academic Health Science Ctr, UK; 24 Manchester Univ NHS. Foundation Trust, Manchester Academic Health Science Ctr, UK; 25 Dept of Clinical Epidemiology & Biostatistics, McMaster Univ, Hamilton ON, Canada; Extramural Rsrch & Internal Portfolio (VP-ERIP), Cape Town, South Africa

**Keywords:** Rheumatic Heart Disease (RHD), Genome-wide association studies (GWAS), African populations, Cardiovascular disease, Group A β hemolytic streptococcus (GAS), Rheumatic heart disease, Rheumatic fever, Cardiovascular disease, Bacterial infections, Group A β hemolytic streptococcus (GAS), Pharyngitis, Genome-wide association study (GWAS), Genetic susceptibility, Genetic epidemiology, Heritability, Genetics, African populations, and Human Hereditary and Health Africa (H3Africa)

## Abstract

**Background:**

The genetics of rheumatic heart disease (RHDGen) Network was developed to assist the discovery and validation of genetic variations and biomarkers of risk for rheumatic heart disease (RHD) in continental Africans, as a part of the global fight to control and eradicate rheumatic fever/RHD. Thus, we describe the rationale and design of the RHDGen study, comprising participants from eight African countries.

**Methods:**

RHDGen screened potential participants using echocardiography, thereafter enrolling RHD cases and ethnically-matched controls for whom case characteristics were documented. Biological samples were collected for the purposes of conducting genetic analyses including a discovery case-control genome-wide association study (GWAS) and a replication trio family study. Additional biological samples were also collected, and processed, for the measurement of biomarker analytes and the biomarker analyses are underway.

**Results:**

Participants were enrolled into RHDGen between December 2012 and March 2018. For GWAS, 2,548 RHD cases and 2,261 controls (3,301 female [69%]; mean age [SD], 37 [16.3] years) were available. RHD cases were predominantly Black (66%), Admixed (23%), and other ethnicities (11%). Among RHD cases, 34% were asymptomatic, 26% had prior valve surgery, and 23% had atrial fibrillation. The trio family replication arm included 116 RHD trio probands and 232 parents.

**Conclusions:**

RHDGen presents a rare opportunity to identify relevant patterns of genetic factors and biomarkers in Africans that may be associated with differential RHD risk. Furthermore, the RHDGen Network provides a platform for further work on fully elucidating the causes and mechanisms associated with RHD susceptibility and development.

## Nonstandard Abbreviations and Acronyms

CRF: Case report form
DOB: Date of birth
GAS: Group A β hemolytic streptococcus
GWAS: Genome-wide association studies
H3Africa: Human Hereditary and Health Africa
LMICs: Low- and middle-income countries
NYHA: New York Heart Association
PCA: Principal component analysis
pTDT: Polygenic transmission disequilibrium testing
REMEDY: Global Rheumatic Heart Disease Registry
RHD: Rheumatic heart disease
RHDGen: The genetics of rheumatic heart disease Network
RF: Rheumatic fever
SSA: Sub-Saharan Africa
SWATH-MS: Sequential Window Acquisition of All Theoretical Mass Spectra

## Introduction

Rheumatic heart disease (RHD) is a preventable sequela of rheumatic fever (RF), characterized by permanent heart valve damage^1^. RHD is the leading indication for cardiac surgery in the young (adolescents and young adults) in Sub-Saharan Africa (SSA), which carries a quarter of the global disease burden^1–3^. Worldwide, RHD affects approximately 40.5 million individuals, claiming up to 340,000 lives annually^4^, the majority of whom live in low- and middle-income countries (LMICs). The recognition of RHD as a common cause of heart failure, infective endocarditis, stroke, and maternal and perinatal mortality over the last two decades led to the reinstatement of RHD as a major public health priority^3^. In 2018, the World Health Assembly passed a resolution on RHD mandating a coordinated global response^1, 5, 6^.

RHD’s persistence as a major public health issue in LMICs is attributed to associated risk factors such as the lack of effective RHD prevention, control and elimination initiatives. Additionally, known risk factors for RF (RHD’s prequel’s) e.g., failure to drastically change socioeconomic factors (affecting those living in poverty and overcrowded dwellings), limited use/availability of antibiotics (especially, intramuscular Penicillin G Benzathine), and increased genetic susceptibility^7^ may also play a role. A recent systematic review indicated that RF was heritable, reporting high odds of RF amongst monozygotic twins with an estimated heritability of 60%^8^. Furthermore, 60% of RF patients develop RHD, and despite there being a proven association between group A *Streptococcus* infection and RHD, the triggered autoimmune process in RHD can occur autonomously after removing the stimulus. This suggests that after initiation of the autoimmune response via molecular mimicry, host factors, most likely genetic, play an important role in disease progression in susceptible individuals^9–11^. Thus, employing genetic studies, which could identify people at high risk, may help develop effective RHD prevention/control measures such as targeted approaches for RHD screening and treatments^8, 12^. Utilizing a hypothesis-free approach, such as a genome-wide association study (GWAS), may highlight key genetic risk factors associated with disease susceptibility^13^.

Here, we report on the rationale and design, and present baseline characteristics, of the RHDGen study with the primary objective to identify genetic variants affecting susceptibility and resistance to RHD in Africans. The secondary objective is to convey the polygenic nature of RHD susceptibility, its heritability in the study population and to replicate any prior or novel GWAS findings. Whilst seeking to identify genetic risk factors associated with RHD, and elucidate the pathogenesis of RHD susceptibility in Africans, a biorepository was formed, that will also serve as a resource for further studies, including biomarker analyses.

## Methods

**Figure 1:**
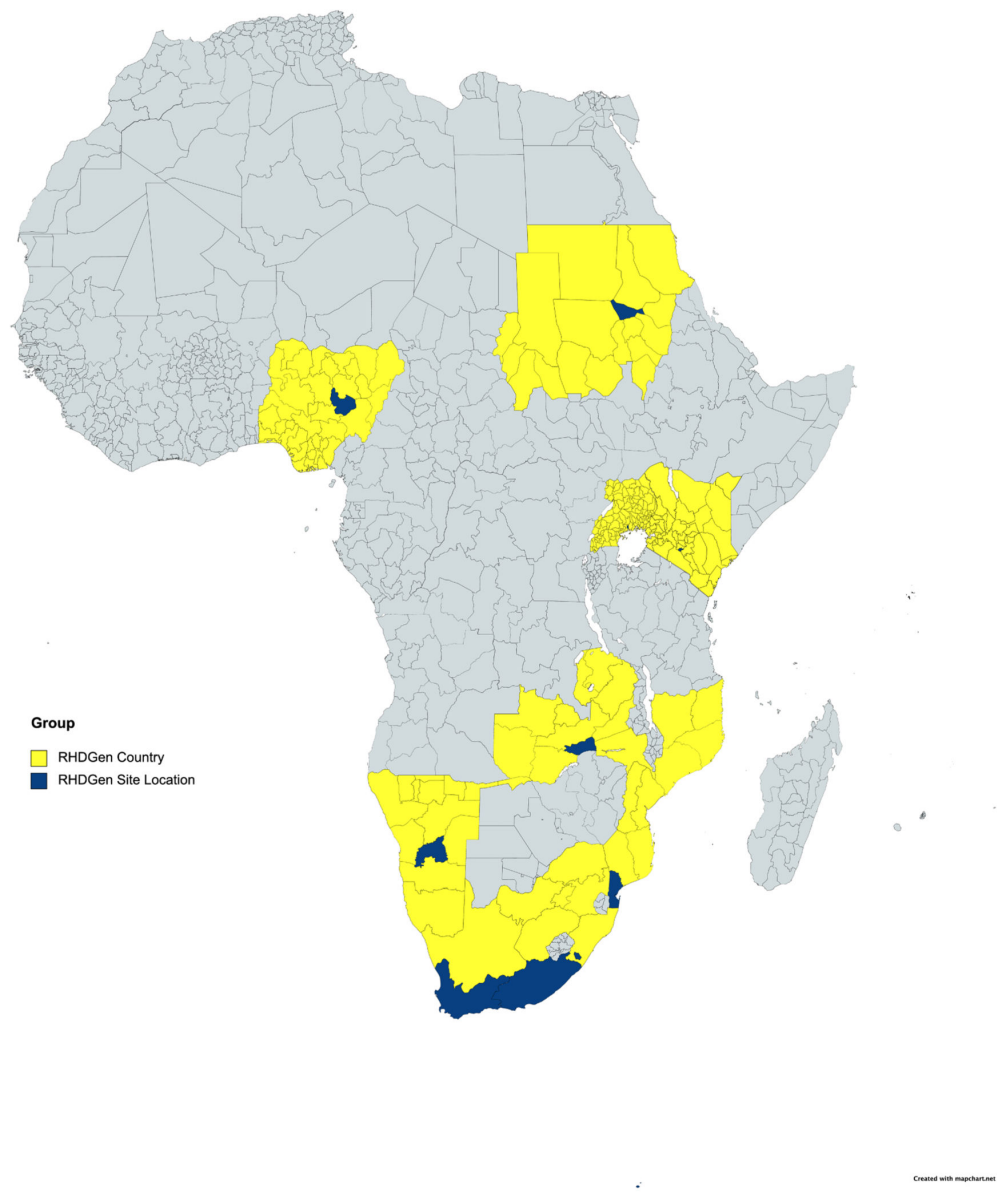
Location of the RHDGen recruitment site countries (yellow) and regional sites (dark blue).

The development of the Rheumatic Heart Disease Genetics (RHDGen) Network (participating countries and sites: Figure 1, Table 1 & 2) and its related sub-studies was approved by appropriate institutional review committees, and all subjects provided written informed consent. Full details of data and methods used in this study are presented in the Supplemental Material and Methods. As per the American Heart Association’s Transparency and Openness Promotion (TOP) Guidelines, we declare that upon reasonable request data will be made available. Due to the sensitive nature of the data collected for this study, reasonable requests to access the dataset from qualified researchers trained in human subject confidentiality protocols may be sent to mark.engel@uct.ac.za and cc: taffymach@yahoo.com. The authors declare that all other supporting data are available within the article and the Supplemental Material.

## Results

### Study Timeline and Baseline Clinical Characteristics

**Figure 2:**
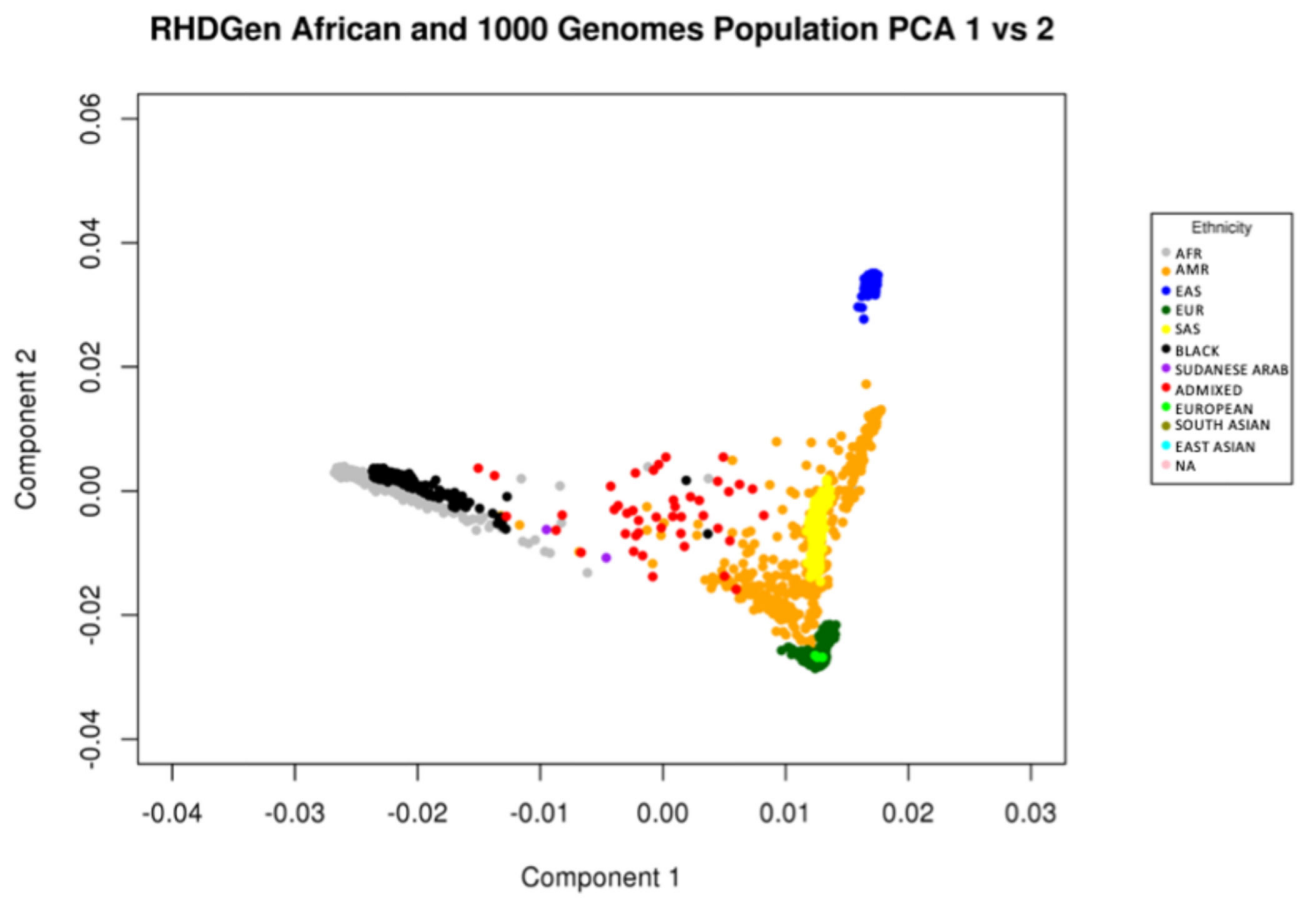

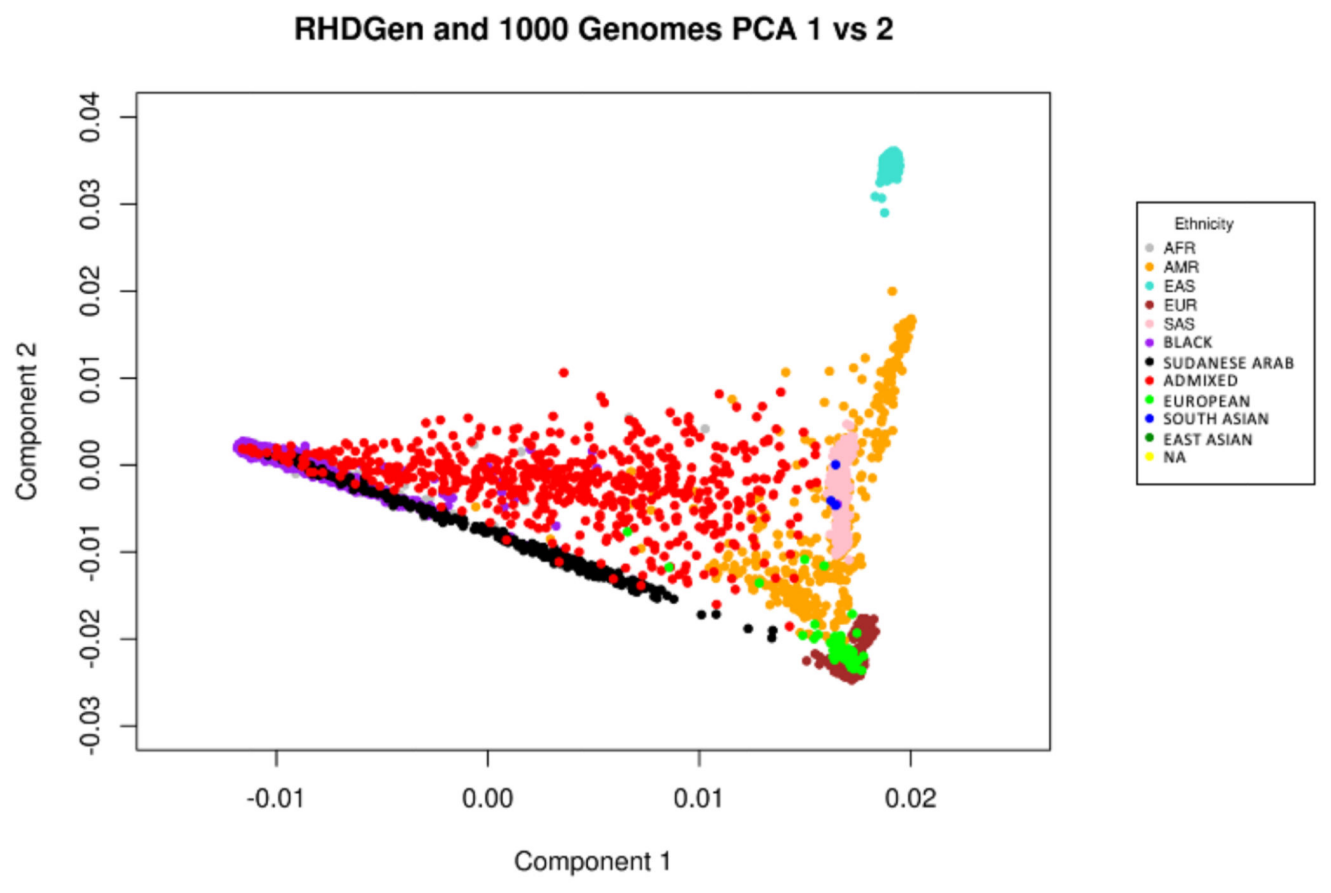
An example of the principal component analysis (PCA) from GCTA for the participants of the RHDGen GWAS and the trio family study. NB:1000G reference populations were from AFR (Black and African American), AMR (Ad Mixed American), EAS (East Asian), EUR (European) and SAS (South Asian) ancestries. From the eight Sub-Saharan African countries in RHDGen, all populations self-reported as citizens/permanent residents of an African country. Hence, the ‘BLACK’ group represents Black Africans, ‘ADMIXED’ represents the ‘South African Coloureds’ (SAC) .i.e. the same as the ‘Admixed Africans’ description, ‘SUDANESE ARAB’ are those who reside in Sudan and self-report being of Arab descent/ethnicity, ‘EUROPEAN’ are those of European ancestry, ‘SOUTH ASIAN’ are those of South Asian ancestry, ‘EAST ASIAN’ are those of East Asian ancestry, and ‘NA’ are those who did not self-report as identifying with any ethnicity/known ancestry.

From December 31, 2012 to March 31, 2018, all GWAS participants and trio probands were screened by echocardiography. Preliminary data cleaning and analyses commenced May 15, 2017, until June 30, 2020. The GWAS arm included 2,548 RHD cases and 2,261 controls (3,301 female [69%]; mean [SD] age, 37 [16.3] years), as per Table 3. RHD cases recruited were predominantly Black (66%), Admixed (23%), and other ethnicities classified as continental African citizens (11%); principal component analysis (PCA) presented in Figure 2A. Baseline characteristics which included 34% of cases were asymptomatic, 26% of cases had valve surgery, and 23% of cases had atrial fibrillation. Most cases (41%) had a slight limitation in physical activity categorized by the New York Heart Association (NYHA) functional classification (Class II); presenting with mild symptoms (mild shortness of breath and/or angina) and slight limitation during ordinary activity^14^. The trio family-based arm included 116 RHD trio probands and 232 matching parents; from the different ancestries across the eight SSA countries in RHDGen (Figure 2B).

### Incidental Outcomes and Benefits

The RHDGen Network was part of the ASAP programme for ARF/RHD in Africa which incorporates awareness-raising as one of four pillars to succesful eradication of RHD from Africa^15^. Thus, fundamental to the RHDGen project, is an overarching commitment to addressing the needs of this patient community in the different countries in which the research took place. Thus, a crucial component of our work is research aimed at making an impact as driven by the African Union communique^16^.

Thus far, the RHDGen Network has also developed additional indirect patient and community benefits, including increased access to RHD screening and patient care, developed and participated in patient/engagement events .e.g., ‘Listen to your heart’ ^17^, hosted disease/genetics educational awareness activities, trained several experts in the field, as well as, advocated globally to change RHD from a neglected tropical disease status to a global health priority. In particular, our work on this and other RHD-related research project has allowed us to work with WHO Africa to revise treatment guidelines for RHD in Africa. Furthermore, this research has allowed our research teams to engage with the World Heart Federation to lobby for greater focus on RHD and other heart conditions prevalent in the Global South.

## Discussion

RHDGen is one of the largest case-control genetic association studies for RHD to date. It is also one of the first studies to evaluate the genetic susceptibility of RHD in continental Africans. While other genetic association studies have examined RHD in four other populations (Aboriginal Australians, Asians, Europeans and Ocenians^18–20^), RHDGen is the first to do so in multiple African countries at large scale^21^.

RHDGen had several unique and versatile features in its rationale, design, and execution. First, a resourceful aspect of this study was that the case report form (CRF) was developed to include the majority of fields from other large RHD studies, like REMEDY (Global Rheumatic Heart Disease Registry). This allowed both current and future clinical/epidemiological and genetic questions to be addressed in the current study, as well as further research, such as meta-analyses with other epidemiological studies. Second, the presence of REMEDY IDs and participants may allow future prospective case analyses, as both participants are screened during REMEDY and RHDGen, as well as retrospective case-control analyses can be performed and genetic factors explored, too. Third, the transfer of the paper-based CRF to the electronic CRF in OpenClinica helped with real-time access to data and actionable visualizations, which made the dataset cleaner and improved workflows. Subsequently, another resourceful aspect of this study was the development of the RHD biobank, as it is a useful resource for future RHD work in Africans. Finally, another innovative aspect of this study is to use family-based genetic data for replication to minimize false positive rates.

RHDGen had several context-specific challenges due to using a family-based design for replication. Although family studies were previously the gold standard for genetic research due to their robustness, in practice they are now rarely used. This is due to the various challenges associated with recruiting complete families, often leading to smaller sample sizes than targeted^22^. Similarly, RHDGen’s trio family recruitment numbers were significantly lower than anticipated; with only 440 families before QC (including parent-child duo families) versus the anticipated 2,000. In our study across eight different African countries, most issues arose from missing parental information.

Key factors that made complete trio recruitment difficult in RHDGen were: First, the African country with the largest GWAS recruitment numbers (i.e. South Africa), had mostly female-headed households (57%) and elevated father absenteeism (~14%) from a variety of reasons including possibly the high adult deaths from HIV/AIDs, since the 1980s. Second, trio probands were estranged from parents who worked as ‘migrant workers’, due to the impacts of the ‘migrant labour system’ e.g., in South Africa under apartheid (1994), which prohibited male pass-controlled laborers from cohabiting with their spouse/child in work-related housing. This has been partially maintained in low income occupations, promoting offspring and working parent(s) estrangement/absenteeism^23^. Third, distances between adult trio probands and their parents’ homes were far or inaccessible, significantly reducing dual parental recruitment. For instance, public health hospital systems and structures (e.g., cardiac clinics) are often centralized and near richer neighbourhoods (including even more private cardiac facilities), as CVD used to be thought to commonly affect only the rich. However, nowadays CVDs like RHD often occur in the children of the poor, who often live further away in impoverished, overcrowded townships/farm areas.

Eventually, towards the end of the study a few community outreaches were attempted to increase trio family member recruitment. For example, Cape Town developed mobile cardiac clinic trips at Helderberg to increase trio recruitment. This outreach was a weekend mobile clinic site and a community-based outreach, enabling complete trio families to provide consent, be screened, provide blood, and be enrolled/included, all at once. Ultimately, the parents were mostly recruited in the countryside (i.e. rural or farming areas), hence, these are poorly-resourced areas where improvisation was needed and limited data were available. Hence, a polygenic transmission disequilibrium testing (pTDT) was employed for replication, to resolve the small sample size and limited parental information available^21, 24^. Thus, future family studies in Africa are recommended to include relevant contextual/tailored financial, transportation and logistics considerations flexible enough to maximize the desired enrollment of the study participants.

### Future directions

We plan to carry out genetic and non-genetic follow-ups for further validation and replication with RHDGen; for instance, attempting collaborative meta-analyses, and mendelian randomization. Future research initiatives in RHD research are recommended to include functional validation through gene disruption studies, fine-mapping, and additional genotyping. Furthermore, a subset of RHDGen serum samples are currently undergoing SWATH-MS evaluation, which will represent the first RHD proteomic profiles in continental Africans. Thus, we invite investigators and funders with shared interests and resources to join and collectively enhance research efforts in RHDGen.

## Conclusion

In summary, the RHDGen Network is a global collaboration among investigators who have recruited patients with RHD across Africa, seeking to gain a better understanding of RHD susceptibility, pathogenesis, and disease development. The RHDGen Network developed a unique network and biorepository to investigate RHD genetics and biomarkers in continental Africans. Ultimately, we hope that our work and collaborations can guide future RHD diagnostic, prevention, management and treatment options.

This research was funded in whole, or in part, by the Wellcome Trust [099313/B/12/A]. For the purpose of open access, the author has applied a CC BY public copyright licence to any Author Accepted Manuscript version arising from this submission.

## Supplementary Material

Supplemental Material

## Figures and Tables

**Table 1:** Participating centres for RHDGen and scheduled activities.

Participating Centre	Ethics	Recruitment of RHD cases and controls	Archiving of data and biological material for local study	Clinical and laboratory graduate training	Training: GWAS technique and analysis	DNA Microarray Assay	SWATH-MS	Bioinformatic processing	Statistical analysis	Archiving of data and biological material for whole study
University of Cape Town (with satellite/mobile centres in Helderberg, Western Cape and Port Elizabeth, Eastern Cape, SA)	**X**	**X**	**X**	**X**				**X**	**X**	**X**
University of Nairobi, Nairobi, Kenya		**X**	**X**	**X**						
Eduardo Mondlane University, Mozambique		**X**	**X**	**X**						
Institute Nacional de Saúde Ministério da Saúde – Moçambique		**X**	**X**	**X**						
Windhoek Central Hospital, Namibia		**X**	**X**	**X**						
Jos University Teaching Hospital, Jos, Plateau State, Nigeria		**X**	**X**	**X**						
AlShaab Teaching Hospital, Sudan		**X**	**X**	**X**						
Mulago Hospital, Kampala, Uganda		**X**	**X**	**X**						
University of Zambia, Lusaka, Zambia		**X**	**X**	**X**						
Population Health Research Institute, McMaster University, Hamilton					**X**	**X**		**X**	**X**	
Centre for Life, University of Newcastle-upon-Tyne								**X**	**X**	
University of Manchester, UK							**TBC**	**X**	**X**	

**X** – represents tasks that have been completed.

**TBC** - represents tasks that are underway; to be completed (TBC) in the future.

The eight SSAn RHD cases were recruited from hospitals together with mostly, community-based controls.

Personnel from the University of Manchester and Newcastle University, UK, assisted with training, guidance and advisement.

SWATH-MS abbreviates Sequential Window Acquisition of All Theoretical Mass Spectra.

**Table 2:** RHDGen countries, their principal investigator (PI), and site timelines.

Site	PI (s)	Site initiation Date	Site closed
South Africa	Bongani Mayosi, Mpiko Ntsekhe	Oct 2013	March 2018
Zambia	John Musuku	Sept 2014	March 2018
Nigeria	Fidelia Bode-Thomas	Sept 2014	March 2018
Namibia	Christopher Hugo-Hamman	Oct 2014	March 2018
Kenya	Bernard Gitura, Stephen Ogendo	Feb 2015	March 2018
Sudan	Ahmed El-Sayed	Feb 2015	March 2018
Uganda	Peter Lwabi, Emmy Okello	May 2015	March 2018
Mozambique	Ana Mocumbi, Albertino Damasceno	May 2015	March 2018

South Africa and Mozambique incorporated additional sites located in Port Elizabeth and Maputo, respectively.

**Table 3:** Clinical characteristics of GWAS participants

Study Participant Profile and Medical History	Cases	Controls
No. of participants (%)	2,548 (53)	2,261 (47)
Female (%)	1,912 (69)	1,389 (75)
Age, years (±SD)	38 (18.8)	36 (13.5)
***Ethnicity (%)***		
Black African	1,687 (66)	1,492 (66)
Admixed African	601 (24)	454 (20)
Other African	260 (10)	315 (14)
***Anthropometric traits (±SD)***		
Weight, kg	62.6 (21.2)	71.4 (18.4)
Height, cm	159.2 (16.9)	164.7 (13.7)
BMI, kg m^-2^	26 (6.1)	27 (6.4)
***Blood pressure related traits (±SD)***		
Diastolic blood pressure, mm Hg	73 (18.5)	77 (11.3)
Systolic blood pressure, mm Hg	118 (20.1)	125 (17.1)
Pulse rate, beats/minute	81.4 (17.1)	75.1(12.8)
***Case specific traits***		
***New York Heart Association (NYHA) Functional Classification (%)***		
NYHA Class I	817 (32)	
NYHA Class II	1,038 (41)	
NYHA Class III	236 (9)	
NYHA Class IV	48 (2)	
No NYHA class listed or unable to determine	409 (16)	
***Valvular regurgitation (%)***	***4,240 (100)***	
Aortic	961 (38)	N/A
Mitral	1,535 (60)	N/A
Pulmonary	373 (15)	N/A
Tricuspid	1371 (54)	N/A
***Valvular stenosis (%)***	***1,096 (100)***	**-**
Aortic	195 (8)	N/A
Mitral	869 (34)	N/A
Pulmonary	6 (0.2)	N/A
Tricuspid	26 (1)	N/A
***Co-occurence (%)***	***1,696 (100)***	**-**
Atrial fibrillation (AF)	578 (23)	3 (0)
Pulmonary hypertension (PHT)	76 (3)	5 (0)
Left ventricular hypertrophy (LVH)	137 (5)	11 (0)
Pregnant	47 (2)	N/A
Asymptomatic cases	858 (34)	N/A
***Treatment Strategies/Interventions (%)***	***3,201 (100)***	**-**
Valve surgery (received prosthetic valve(s))	653 (26)	N/A
Currently on secondary prophylaxis	1,493 (59)	N/A
Never had secondary prophylaxis	758 (30)	N/A
Not reported	297 (11)	N/A

BMI, body mass index; GWAS, genome wide association study; mmHg, millimeters of mercury; NYHA, New York Heart Association; SD, standard deviation.

The continuous variables are given as a mean (±SD). The weight values used to calculate BMI in Table 1 excluded weights >100 kg. NB: Without excluding outliers, R’s MICE package for missing data was used for the complete dataset, including outliers with >100 kg and abnormal height values. The unadjusted average BMI of ‘healthy’ controls was up to 38; this suggests that ‘healthy’ women from our eight African sites were often obese or often overestimated their self-reported weight.

Twenty-six percent of the cases exhibited severe RHD (proposed by severe valvular damage), which warranted the surgical receipt of prosthetic valves.

More than one type of valve and type of valvular damage reported may be present per case. These are overall averages for RHDGen.

Asymptomatic cases were participants recruited after they had an effective surgical or other treatment intervention. The had returned to the clinic for routine follow-up. Hence, they did not present with any common RHD recordable symptoms: e.g., chest pain, dyspnea, fatigue, fever, palpitations and syncope. Only prior medical records, some signs, and echocardiography confirmed their condition.

Valve surgery (received prosthetic valve(s)) means the RHD case previously had surgery and indicated they had at least a prosthetic valve (mechanical and/or bioprosthesis).
