# Supplementary material for: Rationale, Design, and the Baseline Characteristics of the RHDGen (The Genetics of Rheumatic Heart Disease) Network Study
Source: Circ Genom Precis Med. Author manuscript; Available in PMC 2023 Feb 24. (PMC9946164; doi:10.1161/CIRCGEN.121.003641)
Supplement: Supplemental Material [file EMS158255-supplement-Supplemental_Material.pdf]

Supplemental Material

1. Supplementary Methods

1.1 The Rheumatic Heart Disease Genetics (RHDGen) Network

The 2006 Drakensberg declaration, which highlighted and focused continental efforts on raising awareness, providing epidemiological surveillance (e.g., the Global Rheumatic Heart Disease Registry (REMEDY) network[25]), advocating for public policy change, and developing national/regional primary and secondary prevention programmes (e.g., the Awareness, Surveillance, Advocacy, Prevention (A.S.A.P) Programme for RF/RHD) [25], inspired the development of the RHDGen Network. Embedded within the Human Heredity and Health in Africa (H3Africa, https://h3africa.org/) Consortium, RHDGen sought to explore African disease genetics[26-30] with the overarching aim of identifying RHD susceptibility loci in Africans. RHDGen serves to further strengthen previous successful research and collaboration efforts amongst African cardiologist networks, train young African scientists, and maintain the first African RHD biobank with the largest selection of RHD cases globally for genetic association.

1.2 Participating Centres

Several institutions contributed to the network (**main manuscript Table 1**).

1.3 Sampling Framework

RHDGen included eight SSAn countries (**main manuscript Figure 1** and **Table 2**) with adult RHD cases that met RHDGen's inclusion criteria.

During the study designing phase for the GWAS, the target sample size we estimated using QUANTO that we required 3,000 cases and 3,000 controls at an effect size of at least 1.35,

for a lead SNP with minor allele frequency (MAF) > 0.2, using logistic regression models. NB: We ultimately recruited a sample size that met our recruitment plans.

1.4 Ethical Considerations

1.4.1 Informed Consent

Our study included a project bioethicist, JdV, embedded within the RHDGen project to address ethical issues. We used a broad consent model seeking consent for the primary study, sharing, and future use, of data. Consent for future studies carries certain restrictions on the nature thereof and on the decision processes for allowing such studies to take place[25, 31, 32].

1.4.2 Ethics and participant informed consent

JdV developed the informed consent form in conjunction with site principal investigators. All participants gave written informed consent before undergoing evaluation and testing. This study protocol was approved by the University of Cape Town (UCT) Human Research Ethics Committee (*REF: HREC 310/2013*).

The informed consent materials were developed in English, translated to the major local languages and – where local ethics committee guidance required this – back-translated to ensure no loss of original meaning. The RHDGen team also compiled a collection of images to support the consent process and study explanations – including images that demonstrate the study procedures[33]. It was up to recruiters to decide whether and which images to use when describing the study to prospective participants. Since the African continent is diverse and has an excess of 1,000 languages from the Indo-European, Afro-Asiatic, Niger-Congo and Austronesian family of languages[34], our model (in English or in an approved local language translation) accommodated all multinational sites from urban to rural because most African countries teach and practice multilingualism[35].

1.5 Recruitment Procedures

1.5.1 Case Report Form/Case Record Form (CRF)

Our study collected case, trio proband, control and trio parent data, using one of the three types of RHDGen CRFs (**Supplementary Figures I-III**). Each CRF captured essential recruitment data gathered from close-ended questions and checklists, tailored for study participant inclusion or exclusion. We developed our CRFs to be similar to those of an earlier study i.e. REMEDY[25], to make our data compatible and comparable with other available large epidemiological RHD studies.

All case, control and trio proband material covered the expected diagnostic output based on the 2012 World Heart Federation (WHF) criteria for echocardiographic diagnosis of RHD[36]. The trio parent CRF was tailored for trio parent (pseudocontrol) data collection from basic clinical screening. Prior participation in other research studies, co-morbidities and RHD risk factors (if measured) were recorded in the CRF.

1.5.2 Study inclusion criteria

Patients with a primary diagnosis of echocardiographically-confirmed RHD seen in outpatient clinics, emergency departments, and inpatient facilities of participating hospitals were eligible for inclusion as cases. Screening was undertaken among patients with disorders that were specifically known to be due to RHD or that co-presented with RHD, for example, atrial fibrillation and pulmonary hypertension[25]. Controls were participants without a primary diagnosis of RHD and were recruited through advertisements and word of mouth. For trio parent registration, we included parents of participants with a primary diagnosis of RHD (echocardiographic) seen in outpatient clinics, emergency departments, and inpatient facilities of participating hospitals. We also had specific laboratory-related criteria for exclusion described in

Section 1.5.3 and an example illustrated in a schematic diagram of the clinical and laboratory inclusion and exclusion criteria, seen in **Supplementary Figure IV**.

1.5.3 Study exclusion criteria

Patients were excluded from the study if they have the following:

i. A primary diagnosis of valvular disease other than RHD (e.g., degenerative disease).

ii. No informed consent.

iii. No CRF.

iv. No blood sample collected.

v. No DNA from the collected sample (e.g., failed the DNA extraction phase).

CRF data was entered into a bespoke database created with OpenClinica v3.4.1 (an open-source software for Electronic Data Capture and Clinical Data Management), which optimized the management of the clinical data and workflow, smartly and securely[37]. RHDGen recruited 6,354 participants and successfully enrolled 6,253 participants; made up of 3,028 RHD cases (including trio probands), 2,785 controls, and the 314 parents of the successful 157 trio probands. Thereafter, blood samples were collected and pre-processed at respective collection sites, before having DNA extracted, as described by Machipisa *et al* (2021)[38] and the STROBE (Strengthening the Reporting of Observational Studies in Epidemiology) workflow in **Supplementary Figure 5**.

1.6 Sample Collection, Logistics, Storage and Availability

The participants in this study were enrolled into the RHDGen clinical phenotyping scheme illustrated in **Supplementary Figure IV & V**. Each participant was required to provide a minimum of five blood tubes, that were transported to the cardiovascular genetics (CVG) laboratory at the Hatter Institute for Cardiovascular Research in Africa (HICRA) and Cape Heart

Institute (CHI), UCT, South Africa for processing. Four tubes were centrifuged to separate the red blood cells from the white buffy coat layer with plasma or serum by manual pipetting. The fifth blood sample was collected in a PAXgene tube (for long-term storage) for DNA extraction, as per PAXgene DNA extraction kit protocols[39-43].

To ensure sufficient DNA for extensive molecular testing, an extra white buffy coat layer was also separated into two 1.5ml Eppendorf tubes. All blood samples underwent blood fractionation into the relevant component parts, within the recommended timelines as per the BD protocols[39] and the products frozen within 48 hours of collection. Furthermore, all separated components were stored at the recommended temperatures for future use.

For distant sites (>50km away from the CVG lab), plasma, serum and buffy were separated and frozen. Furthermore, PAXgene blood samples for DNA extraction were also frozen and temporarily stored to be batch shipped to CVG for storage and/or centralized/standardized DNA extraction. Archived/biobank data is available but is subject to regulations set by agreements with ethics, prior and current protocols/local policies, and the participant's consent type. We used experienced, registered, regional and international couriers that regularly serviced the region to provide the international transportation of biological samples (e.g., providing frozen sample shipping with dry ice). RHDGen also had multiple Material Transfer Agreements (MTAs), for the different sites' needs, regulations and protocol.

1.7 Genomics Laboratory Processing

For the GWAS, we extracted DNA at the CVG, performed QC, diluted samples for exportation to the Clinical Research Laboratory and Biobank (CRLB) - Genetic and Molecular Epidemiology Laboratory (GMEL), PHRI, Canada for genotyping, and biobanked the remaining samples at CVG. We genotyped the DNA samples at CRLB-GMEL using the Illumina Infinium

Human Omni 2.5M-8 bead chips v1.1 and 1.3[38], according to the manufacturer's protocol, and the raw data was transferred to the PHRI servers.

1.8 Data analysis strategy

1.8.1 RHDGen bioinformatic quality control (QC) workflow

We adopted Turner's 2011 recommendations[44] for per-sample and per-marker QC. Furthermore, we also performed the general RHDGen bioinformatic quality control (QC) workflow, as seen in **Supplementary Figure VI**. After QC, association testing was performed[38].

1.9 Rationale and methodology: RHDGen family-based study (trios*)*

1.9.1 Recruitment

We intended to utilize a trio study as a validation tool, which required a smaller sample size than an ordinary GWAS. The samples were recruited and enrolled during the same time as the GWAS samples, using a purpose-designed CRF. Subsequently, our study only separated the trio and non-trio cohorts after all recruitment was completed. Only patient participants willing to inform their families about their condition and include their families were screened to become part of the proband cohort of trio families. Only pertinent data were collected from parents such as name, DOB and relationship to the proband; in addition, blood, plasma and serum samples were collected.

1.10 Trio data analysis strategy

1.10.1 Trio study

In 2012, the planned sample size for replication was calculated around transmission disequilibrium testing (TDT) trio study model. We estimated using QUANTO[45] that with 1,000 trios and 2,000 parents, however recruitment was lower than anticipated. Hence, methods that can use smaller sample sizes were adopted for TDT (e.g., polygenic TDT) and tested.

1.11 Secondary Analyses of RHDGen

1.11.1 Proteomics

SWATH-MS is a data-independent acquisition mass spectrometry technique created to be a reproducible, label-free method for proteomic analyses e.g., from serum samples, with the sensitivity of targeted methods but with increased proteome depth[46,47].

Scripts and processed data for the biomarker study will be made available on the GitHub repository: https://github.com/jyangUK/Rheumatic_heart_disease[48]. The mass spectrometry proteomics data will be available upon reasonable request, limited to the purpose of Rheumatic Heart Disease research, per ethical permissions for our study, and regulated by a Data Access Committee (Chair: ME). Further details on data accessibility are available from the Corresponding Author.

1.12 RHDGen Data Management and Sharing

Genotype and phenotype data underlying our results will be deposited in the H3A BioNet Data Archive, as per the H3Africa guidelines[25,49]. Some additional restrictions on access and usage apply, with much of the dataset restricted to research focused on RHD. Access to specific components of the dataset requires regulatory approval from the country where the samples were obtained.

2. Materials

2.1 Case Report Forms/Case Record Forms (CRFs)

Our study collected case, proband, control and trio parent data, using one of three types of RHDGen case report forms/case record forms (CRFs) (**Supplementary Figures I-III**). Each CRF captured essential study recruitment data gathered from close ended questions and/or or checklists, tailored for study participant inclusion or exclusion. We developed our CRFs to be

similar to the REMEDY study's CRF[25], to make our data compatible and comparable with other large RHD studies.

Supplementary Figure I: Case/Proband CRF (7 pages)

GLOBAL REGISTRY OF RHEUMATIC HEART DISEASE

REMEDY: RhEuMatic hEart Disease registrY

Page 1
Wellcome H3Africa
RHDGen Network

RHDGen Enrolment: Cases

Subject ID

☐☐☐ *Centre #* ☐☐☐☐☐☐ *Subject #*

☐☐☐☐☐☐☐☐

Hospital # (If known)

Gender: M | F

D.O.B. ______________________YY/MM/DD

Subject Initials

☐☐☐ *F M L*

	Ethnicity	Language
Participant		
Father		
Mother		

Is the Patient enrolled in **REMEDY**? No — proceed to **Page 2**
Yes — If yes, only complete section below

REMEDY number: ____________________

Completed follow-up visit (months)	12	24
Diagnosis made by Echocardiogram?	No	Yes
Echo Source documentation available?	No	Yes
ECG done	No	Yes
ECG Source documentation available?	No	Yes

Bar-coded Sticker

Height ______________________ cm (S123-01)

Weight ______________________ kg (S124-01)

Trio Study	Unique Trio Family number
3 — 2<br>1<br>1. Proband<br>2. Mother<br>3. Father	☐☐☐☐☐☐

Person Completing Report: ________________________________

Date: ☐☐☐☐ *year* ☐☐ *month* ☐☐ *day*

For RHDGen Coordinating Centre use	*Awaiting Info*	*Complete*	*Entered*	*Verified*

C105_01_ClinlExam_Cases enrolment_v3.1_Jan_2015.docx_

Enrolment: Non-REMEDY Participants

Subject ID

☐☐☐ *Centre#* ☐☐☐☐☐☐ *Subject #*

Subject Initials ☐☐☐ *F M L*

Visit date: ☐☐☐☐ *year* ☐☐ *month* ☐☐ *day*

1. Measurements:

a) Blood Pressure (SOP125-01) — Systolic ☐☐☐ / Diastolic ☐☐☐ mmHg

b) Pulse rate ☐☐☐ beats/min

c) Weight (SOP124-01) ☐☐☐ . ☐ kg

d) Height (SOP123-01) ☐☐☐ . ☐ cm

2. Status at the current visit:

a) Symptoms: (please mark (X) as appropriate)

☐ Asymptomatic	☐ Chest pain	☐ Routine clinic visit
☐ Dyspnea	☐ Fever	☐ Palpitations
☐ Syncope	☐ Fatigue	☐ Other, Specify: ________________

b) NYHA class → (please refer to facing page for codes)

☐ I ☐ II ☐ III ☐ IV

3. Pregnancy: (For Women Only)

Is this participant pregnant?

☐ No

☐ Yes

Subject ID

☐☐☐ ☐☐☐☐☐☐
Centre# *Subject #*

Subject Initials ☐☐☐
F M L

4. ECG report:

Date: ☐☐☐☐ ☐☐ ☐☐ (*year* *month* *day*)

a) Source documentation available: ☐ No ☐ Yes

b) Rhythm: ☐ Sinus ☐ Atrial fibrillation ☐ Atrial flutter ☐ Other Dysrhythmia

c) Other comments (specify): ____________________

5. Most Recent CXR:

a) Was a chest x-ray (CXR) performed within last 12 months? *(CXR is only required if clinically indicated)*

☐ No → Will you be obtaining a CXR for this participant? ☐ No → *go to section 6.*
☐ Yes → *Complete sections 5.b-g when CXR obtained*

☐ Yes → *Complete section 5.b-g*

b) Date: ☐☐☐☐ ☐☐ ☐☐ (*year* *month* *day*)

c) CXR report available: ☐ No ☐ Yes

	No	Yes
d) Cardiomegaly	☐	☐
e) Pleural effusion	☐	☐
f) Pulmonary edema	☐	☐

g) Other comments (Specify): ____________________

6. Echocardiogram

a) Was an Echocardiogram (ECHO) performed?

☐ No → *If No, indicate expected date of Echo to be performed – note echocardiogram is mandatory*

☐ Yes:

Date of most recent Echo report: ☐☐☐☐ ☐☐ ☐☐ (*year* *month* *day*)

Report available: ☐ No ☐ Yes

Subject ID

☐☐☐ ☐☐☐☐☐☐
Centre# *Subject #*

Subject Initials ☐☐☐
F M L

Valve Lesions

b) Does the patient have prosthetic valves? ☐ No ☐ Yes → If yes, please mark (X) as appropriate:

	Mechanical	Bioprosthesis
Mitral	☐	☐
Aortic	☐	☐
Pulmonary	☐	☐
Tricuspid	☐	☐

c) Has the patient had an annuloplasty? ☐ No
☐ Yes → If Yes, please specify: ☐ Mitral
☐ Tricuspid

d) Mitral Valve	Absent	Present →	Mild	Moderate	Severe
Regurgitation	☐	☐	☐	☐	☐
Stenosis	☐	☐	☐	☐	☐

Please provide gradient information in section i, ii and iii below, if the Mitral valve is **prosthetic or stenotic**:

→ i) MVA: ☐.☐☐ cm^2

ii) End-diastolic gradient: ☐☐☐.☐☐ mmHg

iii) Mean gradient: ☐☐☐.☐☐ mmHg

Calcification	☐ No	☐ Yes
Vegetations	☐ No	☐ Yes

Subject ID

☐☐☐ *Centre#* ☐☐☐☐☐☐ *Subject #*

Subject Initials ☐☐☐ *F M L*

e) Aortic Valve	Absent	Present →	Mild	Moderate	Severe	(please refer to facing page)
Regurgitation	☐	☐	☐	☐	☐	
Stenosis	☐	☐	☐	☐	☐	

Please provide gradient information in section i, ii, iii and iv below, if the Aortic valve is **prosthetic or stenotic**:

→ i) Jet velocity: ☐.☐☐ m/s iii) Mean gradient: ☐☐☐.☐☐ mmHg

ii) Valve area : ☐.☐☐ cm^2 iv) Peak gradient: ☐☐☐.☐☐ mmHg

Calcification ☐ No ☐ Yes

Vegetations ☐ No ☐ Yes

f) Tricuspid Valve	Absent	Present →	Mild	Moderate	Severe
Regurgitation	☐	☐	☐	☐	☐
Stenosis	☐	☐	☐	☐	☐

Please provide doppler gradient information below, if the Tricuspid valve is **prosthetic or stenotic**:

→ Doppler gradients (in mmHg): mean ☐☐☐.☐☐ End-diastolic ☐☐☐.☐☐

Calcification ☐ No ☐ Yes

Vegetations ☐ No ☐ Yes

g) Pulmonary Valve	Absent	Present →	Mild	Moderate	Severe
Regurgitation	☐	☐	☐	☐	☐
Stenosis	☐	☐	☐	☐	☐

Please provide doppler gradient information below, if the Pulmonary valve is **prosthetic or stenotic**:

→ Doppler gradients (in mmHg): peak ☐☐☐.☐☐ mean ☐☐☐.☐☐

Calcification ☐ No ☐ Yes

Vegetations ☐ No ☐ Yes

Subject ID

☐☐☐ *Centre#* ☐☐☐☐☐☐ *Subject #*

Subject Initials ☐☐☐ *F M L*

h) Pulmonary hypertension TR gradient: ☐☐.☐☐ mmHg

TR velocity: ☐☐.☐☐ m/s

i) Left ventricular dimensions LVIDd ☐☐.☐ mm

LVIDs ☐☐.☐ mm

j) Left ventricular ejection fraction: ☐☐.☐ %

k) Left ventricular shortening fraction: ☐☐.☐ %

l) Left atrium AO: ☐☐.☐ mm

LA: ☐☐.☐ mm

LA:AO ratio: ☐☐.☐☐

m) Additional echo findings:

Spontaneous echo contrast ☐ No ☐ Yes

Pericardial effusion ☐ No ☐ Yes

Left atrial thrombus ☐ No ☐ Yes → Details: ____________________
→ Size: ☐☐☐ x ☐☐☐ mm

Thrombi other than LA ☐ No ☐ Yes → Details: ____________________
→ Size: ☐☐☐ x ☐☐☐ mm

n) Further comments: ____

Subject ID

☐☐☐ *Centre#* ☐☐☐☐☐☐ *Subject #*

Subject Initials ☐☐☐ *F M L*

Medication:

7. Secondary prophylaxis

a) Is the participant:

→ Specify:

☐ Currently on secondary prophylaxis
  - ☐ Benzathine penicillin
  - ☐ Oral agents

☐ Past use of secondary prophylaxis

☐ Never

Person Completing Report: ______________________ Name / Signature

Date: ☐☐☐☐ ☐☐ ☐☐

Supplementary Figure II: Control CRF (3 pages)

REMEDY: RhEuMatic hEart Disease registrY

Wellcome H3Africa
RHDGen Network

RHDGen Enrolment : Control

Subject ID

☐☐☐ *Centre #* ☐☐☐☐☐☐ *Subject #*

Subject Initials

☐☐☐ *F M L*

☐☐☐☐☐☐☐☐

Hospital # (If known)

Bar-coded Sticker

	Ethnicity	Language
Participant		
Father		
Mother		

Visit Date: ☐☐☐☐ *Year* ☐☐ *month* ☐☐ *day*

Gender: M | F

D.O.B: ☐☐☐☐ *Year* ☐☐ *month* ☐☐ *day*

1. **Measurements**

a) Blood Pressure (*SOP125-01*) Systolic ☐☐☐ / Diastolic ☐☐☐

b) Pulse rate ☐☐☐ beats/min

c) Weight (*SOP124-01*) ☐☐☐ . ☐ kg

d) Height (*SOP124-01*) ☐☐☐ . ☐ cm

C106_01_ClinlExam_Control enrolment_v0.2.docx

2. 12-Lead ECG Report

Visit Date: ☐☐☐☐ ☐☐ ☐☐
Year month day

a) **ECG Done** N / Y *If **NOT done,** what is the scheduled date* ☐☐☐☐ ☐☐ ☐☐
Year month day

b) **Rate** ☐☐☐ beats/min

c) **Rhythm**
- ☐ Sinus rhythm
- ☐ Atrial fibrillation
- ☐ Other dysrrhythmia (Specify) ____________

d) **Voltages In mm**

i)	SV_1	☐☐.☐	mm
ii)	RV_5	☐☐.☐	mm
iii)	RV_6	☐☐.☐	mm
iv)	RaVL	☐☐.☐	mm
v)	SV_3	☐☐.☐	mm

3. Echo Report

Date of Echocardiogram: ☐☐☐☐ ☐☐ ☐☐
Year month day

Where appropiate please mark (x)

Image Quality: ☐ Good ☐ Average ☐ Poor

a) **Valve Abnormalities** ☐ No ☐ Yes → if Yes, exclude from study.

b) M- Mode Measurements (Take an average of 3 measurements for each parameter).

i) Left ventricular dimensions:

IVSd	☐☐.☐	mm
IVSs	☐☐.☐	mm
LVIDd	☐☐.☐	mm
LVIDs	☐☐.☐	mm
LVPWd	☐☐.☐	mm
LVPWs	☐☐.☐	mm

ii) Left ventricular ejection fraction: ☐☐.☐ %

iii) Left ventricular shortening fraction: ☐☐.☐ %

iv) Left atrium:

AO	☐☐.☐	mm
LA	☐☐.☐	mm

c) Comments: ____

Person completing the report: ________________________________ ☐☐☐☐ ☐☐ ☐☐

Name / Signature *Year* *month* *day*

Supplementary Figure III: Trio Parent CRF (a one-page example of a mother of a proband recruited)

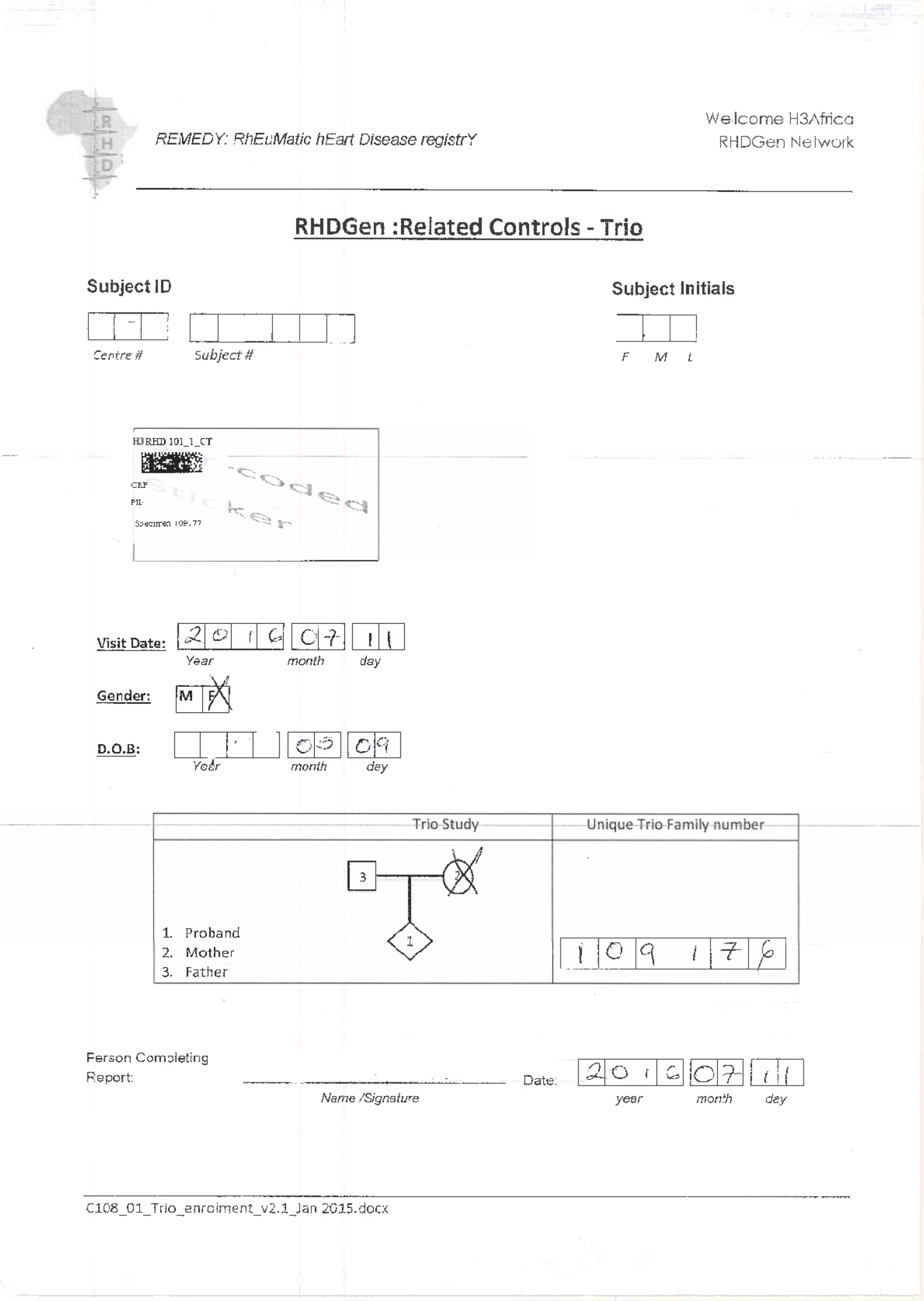

REMEDY: RhEuMatic hEart Disease registrY

Welcome H3Africa
RHDGen Network

RHDGen :Related Controls - Trio

Subject ID

Centre # *Subject #*

Subject Initials

F M L

H3RHD 101_1_CT

CRF

PII-

Specimen 109.77

Bar-coded sticker

Visit Date: 2016 07 11 (*Year* *month* *day*)

Gender: M F (F crossed)

D.O.B: [illegible] 05 09 (*Year* *month* *day*)

Trio Study	Unique Trio Family number
1. Proband<br>2. Mother<br>3. Father	109176

Person Completing Report: ______________ *Name /Signature* Date: 2016 07 11 (*year* *month* *day*)

C108_01_Trio_enrolment_v2.1_Jan 2015.docx

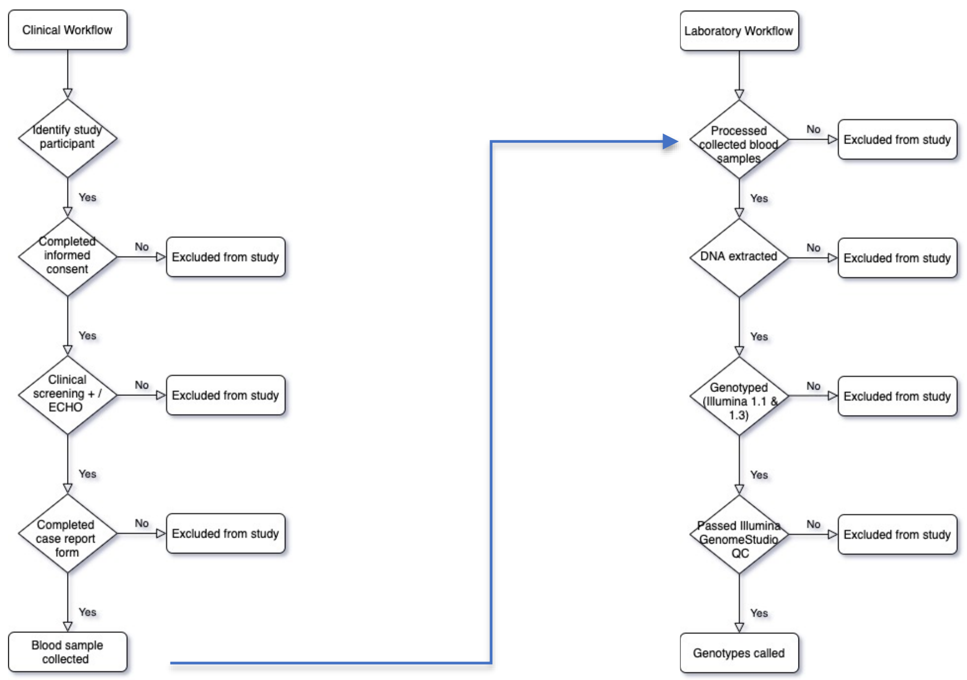

Supplementary Figure IV: RHDGen clinical and laboratory workflow inclusion and exclusion criteria.

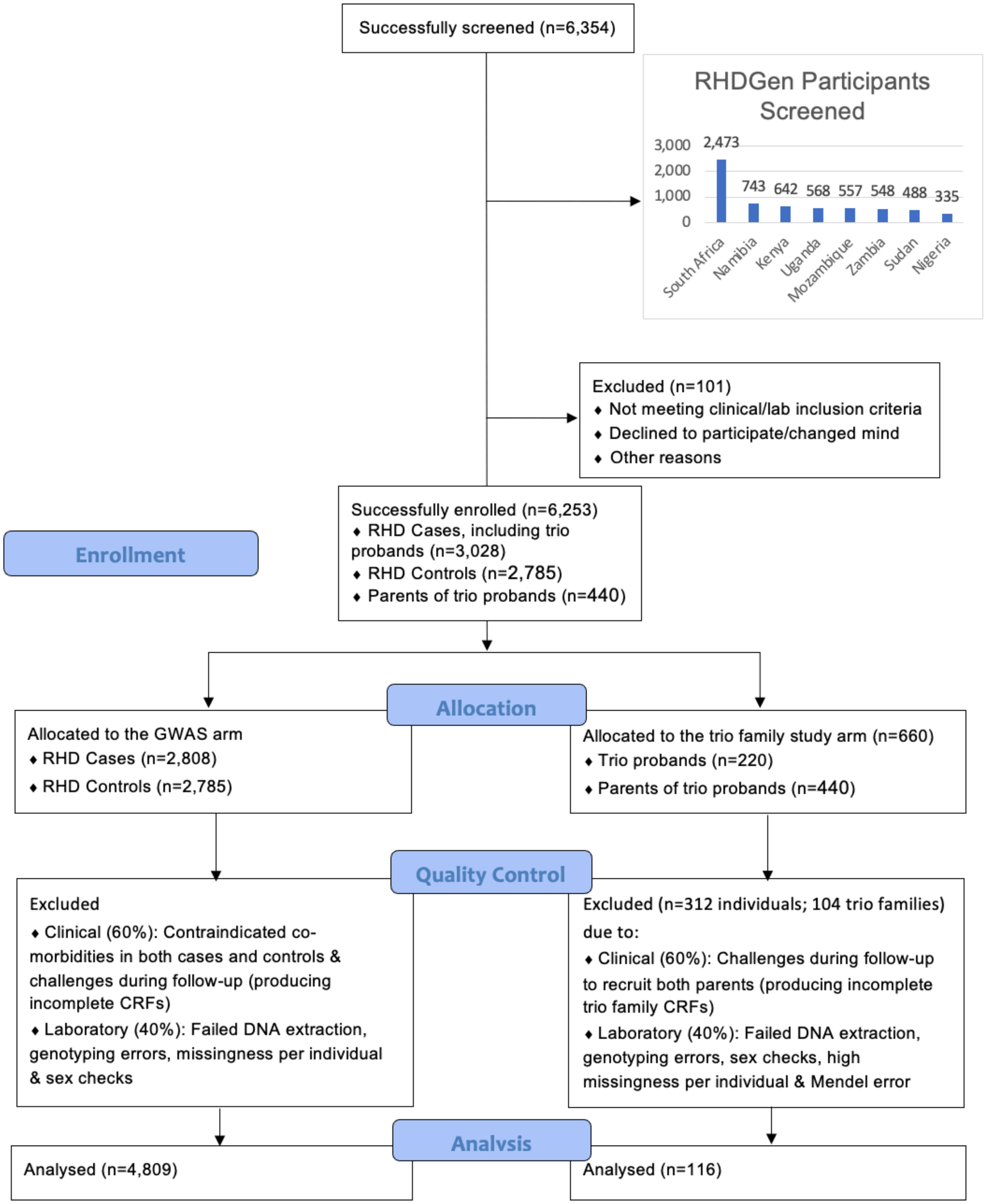

Supplementary Figure V: RHDGen's STROBE (Strengthening the Reporting of Observational Studies in Epidemiology) flowchart.

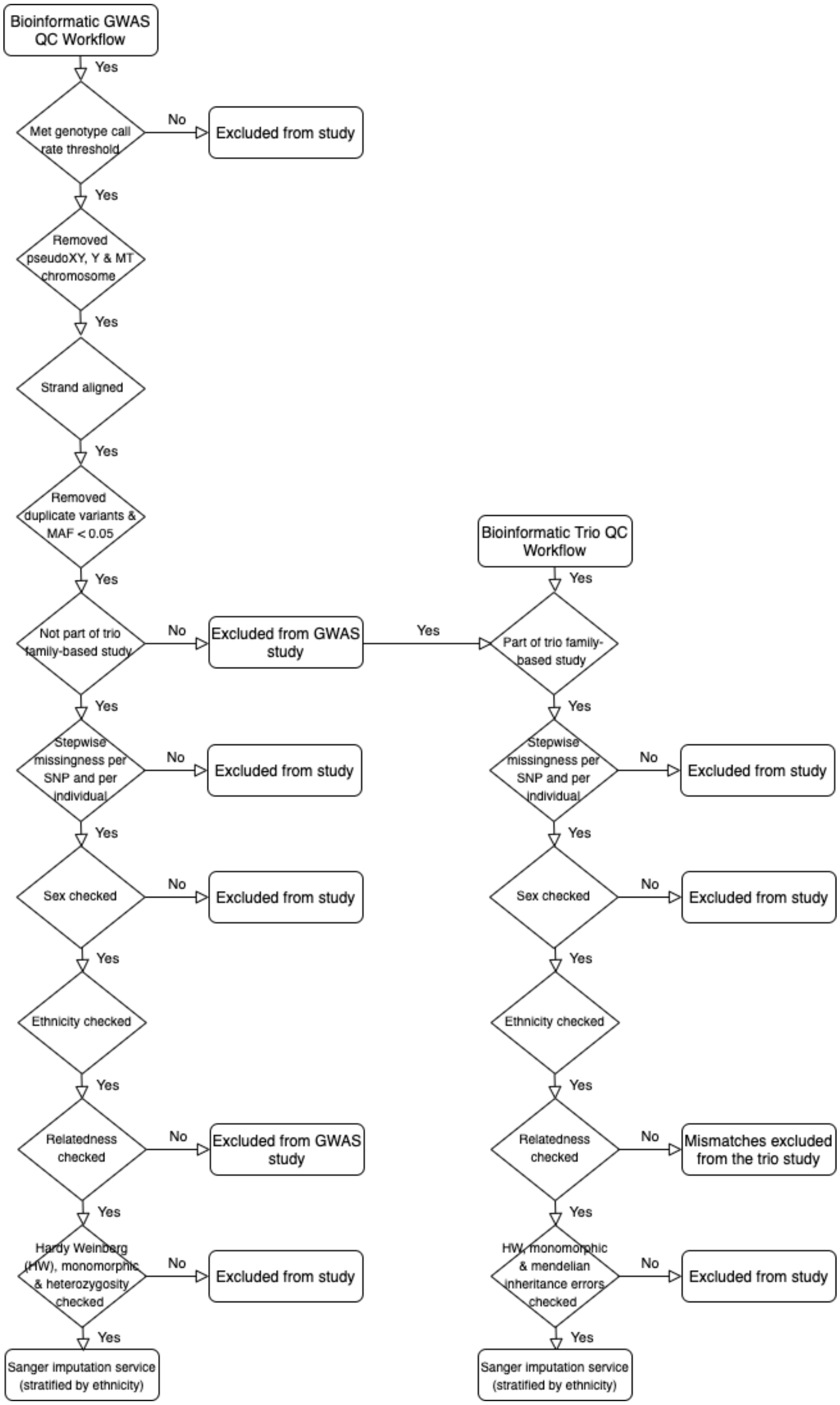

Supplementary Figure VI: RHDGen bioinformatic quality control (QC) workflow.

3. Outcomes

3.1 Incidental Outcomes and Benefits

The RHDGen Network was driven by a group of clinician and medical researchers based at African universities who have dedicated their careers to investigating RHD and addressing the needs of this patient community in the different countries in which the research took place. Thus, a crucial component of our work is research - making sure that data is produced that allows us to understand the impact of this disease on patients and their families, but also understanding better the biology and epidemiology of this disease to inform policies and treatments in future.

Another crucial component of our work is engaging the RHD patient communities that are seen in our hospitals/clinics/study sites. Patient engagement looks different in the many countries that conducted RHDGen; in some countries, it involved organisation of patient awareness events every year; in others, it meant attending to patient needs outside of clinic hours. Nevertheless, across all the countries, the needs of our patient community were central to our work.

In terms of ensuring benefit - although it is difficult to identify concrete ways in which the results of genomic studies can directly benefit patients (because translation of knowledge to the clinic usually takes time), there are indirect ways in which the project described in our manuscript has indeed benefited the RHD patient community. In particular, our work on this and other RHD-related research project has allowed us to work with WHO Africa to revise their treatment guidelines for RHD in Africa. Furthermore, this research has allowed our research teams to engage with the World Heart Federation to lobby for greater focus on RHD and other heart conditions prevalent in the Global South.

THE GENETICS OF RHEUMATIC HEART DISEASE (RHDGEN) NETWORK CONSORTIUM

Founding Senior PI: Bongani M Mayosi, MD DPhil

Study conceptualization/design team (RHDGen grant: 099313/B/12/A): B M Mayosi, H J Cordell, J de Vries, A El Sayed, ME Engel, CT Hugo-Hamman, M Lesosky, B Keavney, A Mocumbi, C Mondo, N Mulder, J Musuku, G Paré, Raj Ramesar, O Ogah, S Ogendo, G Shaboodien, T Machipisa;

INVESTIGATORS, COMMITTEES, AND STAFF

Investigators – number of patients enrolled in each country and site are in parenthesis

South Africa (2473): The Cardiac Clinic, Groote Schuur Hospital, Cape Town (2216) – M Ntsekhe, C Chishala, B Cupido; Livingstone and Provincial Hospitals, Port Elizabeth (257) - L Pepeta; Namibia (743): Rheumatic Heart Disease Clinic, Windhoek Central Hospital, Ministry of Health and Social Services, Windhoek, Republic of Namibia – CT Hugo-Hamman, T Auala, C Brinkman, L Sikwaya; Kenya (642) - Cardiology Department of Medicine, Kenyatta National Hospital, University of Nairobi – Bernard M Gitura, Stephen Ogendo, Tom Omulo; Uganda (568): Uganda Heart Institute, Departments of Adult and Peadiatric cardiology, Kampala, Uganda – P Lwabi, E Okello; Mozambique (557): Faculty of Medicine, Eduardo Mondlane University / Nucleo de Investigaçao, Departamento de Medicina, Hospital Central de Maputo, Maputo, Mozambique (367) - A Damasceno, A Fabula; Instituto Nacional de Saúde Ministério da Saúde – Moçambique (190) – A Mocumbi, G Madeira; Zambia (548): University Teaching Hospital – Children's Hospital, University of Zambia, Lusaka, Zambia - J Musuku, A Mtaja; Sudan (488): Department of Cardiothoracic Surgery, Alshaab Teaching Hospital, Alazhari Health Research Center, Alzaiem Alazhari University, Khartoum, Sudan – A ElSayed, HHM

Alhassan, A Abdelhalim, H Alhajapo, E Idris, S Salih; Nigeria (335): Departments of Paediatrics and Medicine, Jos University Teaching Hospital and University of Jos, Jos, Plateau State Nigeria – F Bode-Thomas, B Okeahialam, S Danbauchi, O Ige, C Yilgwan, G Amusa, E Nkereuwem.

Project Coordinating Office, University of Cape Town, South Africa
ME Engel (Study Manager), R Daniels (Research Assistant), J de Vries (Ethicist), V Francis (Coordinator), F. Gili (Research Assistant), P Kraba (Research Assistant), S Pandie (Data Manager), R. Vergotine (Data Assistant);

Project Coordinating Laboratory, Cardiovascular Genetics, Hatter Institute for Cardiovascular Research in Africa (HICRA) & Cape Heart Institute (CHI), University of Cape Town, South Africa
G Shaboodien (Director), T Machipisa (Laboratory Scientist), B Muhamed (Laboratory Scientist), S Kamuli (Laboratory Scientist), L Pearce (Research Assistant), J Saaiman (Research Assistant);

Bioethics Research Initiative, Department of Medicine, University of Cape Town, South Africa
J de Vries (Director), Research Fellows: M Faure, F Masiye, O P Matshabane, N S Munung;

Population Health Research Institute (PHRI), Hamilton Health Sciences and McMaster University RHDGen Project Team (Clinical Research Laboratory and Biobank - Genetic Molecular Epidemiology Laboratory, CRLB-GMEL)

G Paré (CRLB-GMEL Director), Michael Chong (Bioinformatician), R Ditta (Laboratory Coordinator), A Hodge (Laboratory Technician), T Machipisa (visiting RHDGen Fellow);

Research Assistants, Coordinators, Fieldworkers

South Africa: The Cardiac Clinic, Groote Schuur Hospital, Cape Town – U September (Research Nurse), S Nkepu (Field Site Coordinator), M Van de Wall (Echocardiographer), N Laing (Genetic Counsellor), A Joachim (Research Nurse); Livingstone and Provincial Hospitals, Port Elizabeth (257) – R Solwandle (Research Nurse); Namibia (743): Rheumatic Heart Disease Clinic, Windhoek Central Hospital, Ministry of Health and Social Services, Windhoek, Republic of Namibia – G Olivier (Field Site Administrator), A Awases (Research Nurse), A Bock (Research Nurse), S Nzuza (Clinical Technologist), C Mangami (Clinical Technologist), H Amanyanga (Research Nurse), A Mneumbo (Research Nurse); Kenya (642) - Cardiology Department of Medicine, Kenyatta National Hospital, University of Nairobi – Elizabeth Musyoki (Research Nurse); Uganda (568): Uganda (568): Uganda Heart Institute, Departments of Adult and Peadiatric cardiology, Kampala, Uganda – I Ssinabulya (Clinician Researcher), S Kitoleko (Research Nurse), J Kebba (Labarotory Technologist); Mozambique (557): Faculty of Medicine, Eduardo Mondlane University / Nucleo de Investigaçao, Departamento de Medicina, Hospital Central de Maputo, Maputo, Mozambique (367) - C Novela (Research Coordinator), M Machava (Research Assistant), V Govo (Research Assistant); Instituto Nacional de Saúde Ministério da Saúde – Moçambique (190) – S Candido (Research Nurse); Zambia (548): University Teaching Hospital – Children's Hospital, University of Zambia, Lusaka, Zambia; A Lungu (Research Nurse), T Kaira (Laboratory Technologist); Sudan (488): Department of Cardiothoracic Surgery, Al Shaab Teaching Hospital, Faculty of Medicine, Alzaiem Alazhari University, Khartoum,

Sudan – NKM Elkhder (Radiographer); Nigeria (335): Departments of Paediatrics and Medicine, Jos University Teaching Hospital and University of Jos, Jos, Plateau State Nigeria – C Barau (Research Nurse), OA Marcaulay (Research Assistant), D Badung (Laboratory Scientist), C Durojaiye-Amodu (Data Clerk);
